# Computational Analysis of Histone Deacetylase 10 Mechanism by the ONIOM Method: A Complementary Approach to X-ray and Kinetics Studies

**DOI:** 10.1021/acsomega.1c07055

**Published:** 2022-02-09

**Authors:** Ibrahim Yildiz, Banu Sizirici Yildiz

**Affiliations:** † Chemistry Department, Khalifa University, P.O. Box 127788, Abu Dhabi, United Arab Emirates; ‡ CIVE Department, Khalifa University, P.O. Box 127788, Abu Dhabi, United Arab Emirates

## Abstract

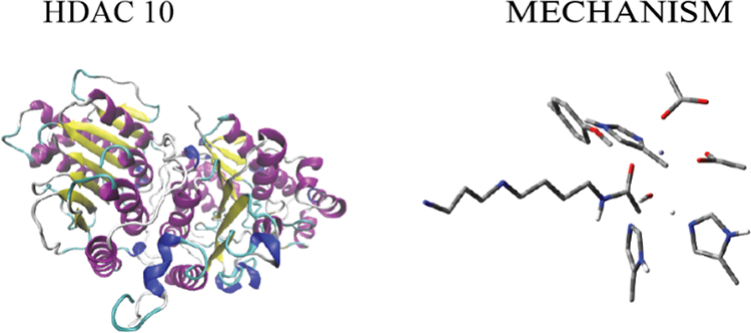

Histone deacetylase 10 (HDAC 10) catalyzes deacetylation of N^8^-acetylspermidine into spermidine in the cytosolic region of eukaryotic cells. Inhibition of HDAC 10 has clinical importance in certain types of cancers. Recently, X-ray crystal structures corresponding to the substrate-bound, tetrahedral intermediate-bound, and product-bound enzymes have been resolved using variant forms of humanized HDAC 10. Based on these structures, it was proposed that Y307 residue polarizes the carbonyl of the acetyl group in N^8^-acetylspermidine together with a zinc atom, which is coordinated by D174, H176, D267, and an H_2_O molecule. The H_2_O molecule undergoes nucleophilic addition to the carbonyl carbon of N^8^-acetylspermidine to form the tetrahedral intermediate. During this process, it is suggested that H136 acts as a general base to deprotonate the H_2_O molecule. It is further proposed that the protonation of the amide N atom of the tetrahedral intermediate by H137 causes the deacetylation forming the final products, spermidine and acetate ion. In this study, computational models based on the ONIOM method were employed to study the proposed mechanism for the two steps of the deacetylation process based on the crystal structure of the substrate-bound enzyme. The energy profiles of each step as well as the roles of the active site residues were investigated for the catalysis. The calculated activation barrier is in good agreement with the reported *k_cat_* value.

## 1 Introduction

**Figure 1:**
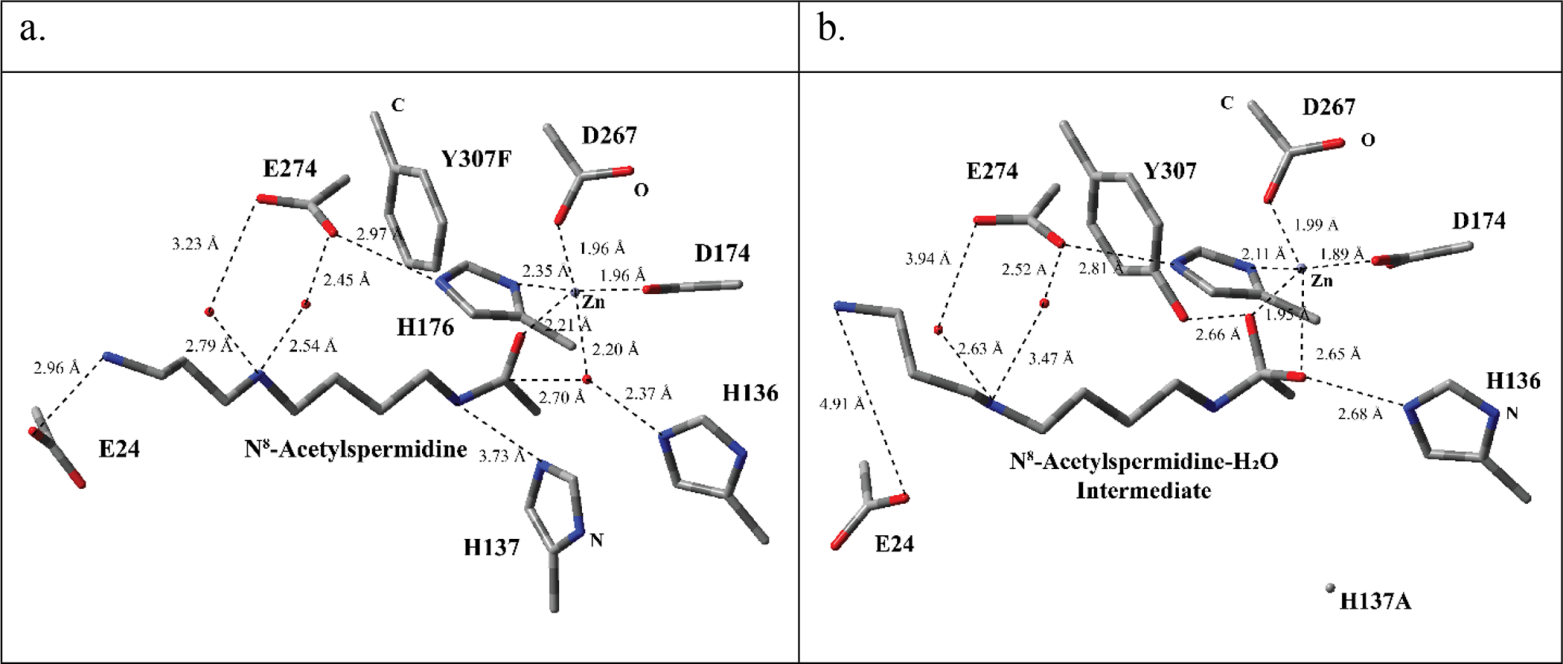
HDAC 10 complexed with (a) the substrate N^8^-acetylspermidine and (b) tetrahedral oxyanion intermediate.

Histone deacetylases (HDAC) are composed of 18 functionally related isozymes.^1^ They catalyze the removal of acetyl groups from fatty-acids,^2^ polyamines,^3^ and fatty-acid lysines.^4^ The selective inhibitors of HDAC may provide therapeutic benefits against cancer, neurological diseases, and immune disorders.^5^ A recent study revealed that HDAC 10 indeed belong to the polyamine deacetylase (PDAC) family based on a glutamate gate-keeper and a constricted active site, which favors hydrolysis of slender polyamines and disfavors hydrolysis of acetyllysines.^3^ Herbst-Gervasoni and Christianson have recently resolved the crystal structures of zebrafish HDAC 10 employing A24E and D94A mutations to obtain a “humanized” variant.^6^ By employing further mutations in the active site on the essential residues for the deacetylation of the native substrate, N^8^-acetylspermidine, they were able to co-crystallize the substrate-bound, intermediate-bound, and product-bound enzymes. In the first instance, the variant Y307F lacked the phenolic −OH group of the tyrosine residue, which is suggested as an essential group to polarize carbonyl oxygen of the N^8^-acetylspermidine for the nucleophilic addition of an H_2_O molecule. Indeed, the crystal structure of this variant provides a snapshot of a substrate-bound active site, which is essentially a non-productive enzyme–substrate complex (a in Figure 1). In the second instance, the variant H137A lacked the histidine residue, which provided the co-crystallization of the enzyme-bound reaction intermediate complex (b in Figure 1). The doubly protonated form of H137 is proposed to act as a general acid catalyst facilitating the amide bond breaking through protonation of amide N forming spermidine and acetate ions.

**Figure 2:**
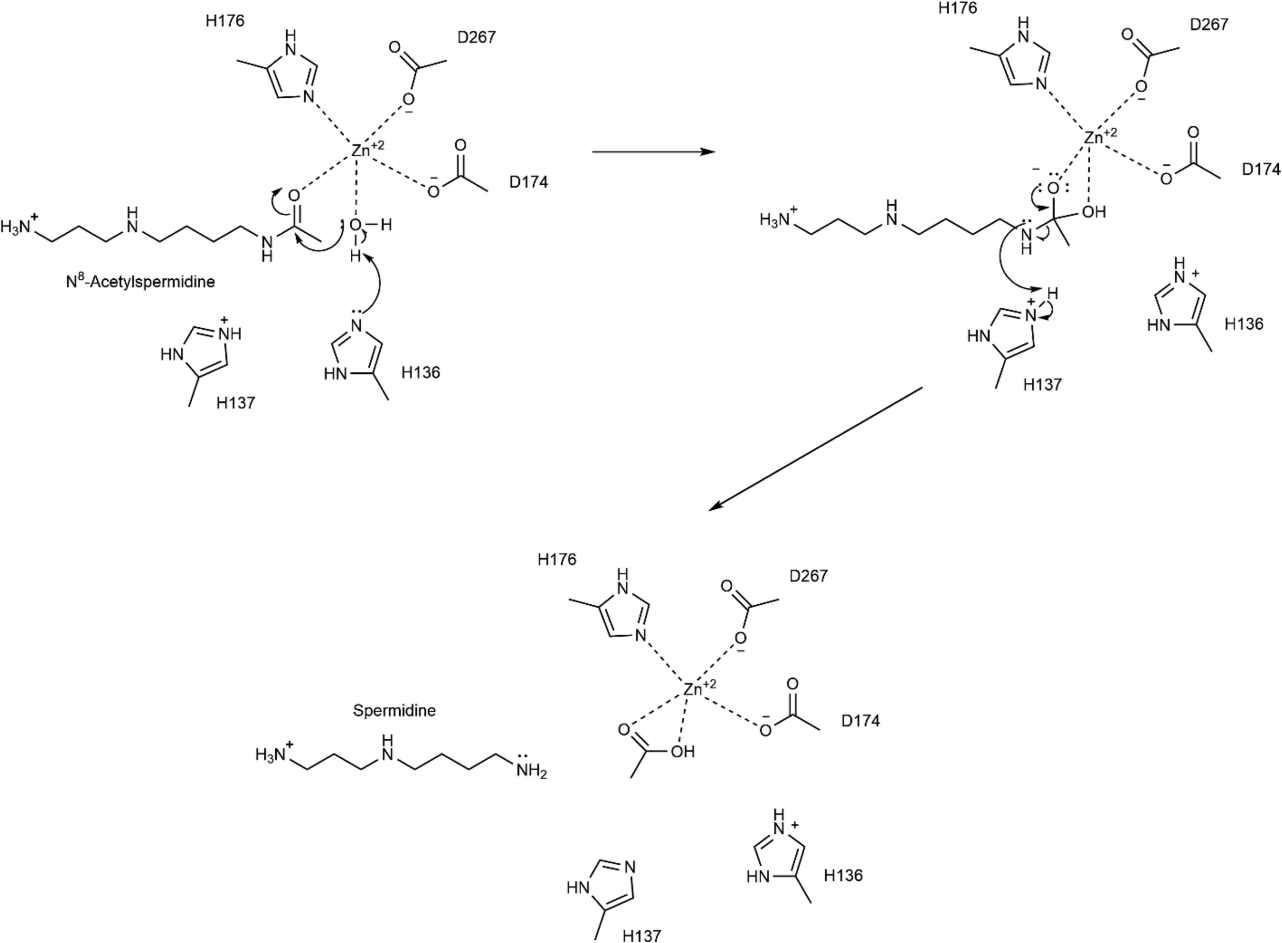
Proposed reaction mechanism for the deacetylation of N^8^-acetylspermidine by HDAC 10.

Based on the resolved crystal structures, the proposed mechanism for the HDAC 10 can be summarized in two main steps. In the first step, a H_2_O molecule in the active site is rendered more nucleophilic through a H-bonding interaction with the H136 residue (Figure 2). Furthermore, the carbonyl C of the amide group in N^8^-acetylspermidine is made more electrophilic through the coordination of Zn^2+^. As a result, the nucleophilic addition of the OH^–^ ion results in the formation of the tetrahedral intermediate and protonation of H136 residue (Figure 2). In the second step, the amide bond breaks as H137 protonates the N atom of the tetrahedral intermediate leading to spermidine, acetate ion, and neutral H137.

The crystal structure of substrate-bound HDAC 10 reveals important interactions in the active site (Figure 1a). The catalytic Zn ion is penta-coordinated with D267, H136, H176, an active site H_2_O molecule, and carbonyl O of N^8^-acetylspermidine. The active site H_2_O molecule has a H-bonding interaction with H136. H137 is 3.73 Å away from the N8 of N^8^-acetylspermidine. In addition to Zn^2+^ coordination, H176 seems to have a H-bonding interaction with E274, which interacts with the N4 of N^8^-acetylspermidine through two H_2_O molecules. E24 is positioned to have a H-bonding interaction with the N1 of N^8^-acetylspermidine. The crystal structure of the tetrahedral intermediate reveals similar active site interactions. The Zn^2+^ atom is still penta-coordinated with the same residues albeit the O atom of the active site H_2_O molecule is now part of the acetate group of the intermediate and it is further away from Zn^2+^. The O atom in Y307 has a close H-bonding interaction with the O atom of the acetate group of the intermediate. One prominent difference between the substrate-bound active site and the tetrahedral intermediate bound active site is that after the formation of the tetrahedral intermediate, the conformation of the polyamine, spermidine, changes from linear to bent. This transformation presumably occurs because of the C3–N4 bond rotation of the spermidine. This suggests that formation of the tetrahedral intermediate is accompanied by some rearrangements in the active site. For example, the distance between the O atoms of the two H_2_O molecules with the O atoms of E274 and N4 of the spermidine are quite different. In addition, the distance between the E24 and N1 positions of the spermidine is almost 2 Å more as compared to the substrate bound active site.

It has been shown that inhibition of HDAC 10 causes improved efficacy of doxorubicin against neuroblastoma cells.^7^ Thus, understanding the details of the HDAC 10 mechanism might offer invaluable information for the treatment of a variety of cancer types.^8^ In general, there are two main proposed deacetylation mechanisms for Zn^2+^ bound HDAC enzymes. In the first instance, a dyad of His residues act as general acid–base catalysts in the deacetylation process in which one of the singly protonated His residue acts as a general base to deprotonate the active site water, and the second doubly protonated His residue acts as general acid to protonate the amide N.^9,10^ This mechanism is also consistent with the proposed mechanism for HDAC 10 (Figure 2). The second mechanism is based on a proton shuttle mechanism in which one of the His residue in the dyad act as both the general acid and base catalyst.^11^ On the basis of results of QM-MM MD simulations and umbrella sampling for HDAC 8, one of the His residue of dyad first deprotonates the active site molecule and then transfers this proton to the amide N of the substrate. Recently, Nechay et al. studied the two mechanisms using the QM/DMD method including different divalent metal ions.^12^ They found that the proton shuttle mechanism is more energetically feasible than the general acid–base mechanism. Furthermore, the rate-limiting step was found to be not the water deprotonation step but the amide N protonation step.

Computational studies for the deacetylation process of various enzymes such as HDAC 8,^11,12^ GCN5,^13^ and HDLP^14^ utilized hybrid QM-MM methods in which the active site together with the substrate is treated with the DFT functional, and the surrounding region is treated with the MM force-field. These calculations provide invaluable information in terms of energetics of the proposed reaction mechanisms and important interactions during the chemical transformations. In this study, the proposed deacetylation mechanism for HDAC 10 was investigated with the ONIOM method consisting of QM-MM calculations using the crystal structure of HDAC 10 complexed with N^8^-acetylspermidine. The possible reaction pathways were studied by means of potential energy surface (PES) scans considering possible protonation states of catalytic His dyad. Based on several different models, enzyme–reactant complexes, the transition states, and model enzyme–product complexes were generated. With the help of these models, the roles of important residues were probed, and the energetics possible pathways were evaluated by calculating activation energies.

## 2 Computational Details and Methodology

A two-layer ONIOM^15^ method was used for the calculations using the Gaussian 09 package.^16^ cam-B3LYP,^17^ M06-2X,^18^ and ωB97XD^19^ were employed in the QM layer. In the MM region, AMBER force field^20^ was used. The B3LYP functional was found to generate complementary results to experimental studies in enzyme reactions; the cam-B3LYP functional has both the hybrid quality of B3LYP and the long-range correction.^21,22^ The M06-2X functional showed better performance in the main-group chemistry with respect to B3LYP.^23^ ωB97XD uses empirical dispersion with long-range corrections. The RESP charges of atoms at N^8^-acetylspermidine were calculated with the HF/6-31G(d) method.^24^ Amber 94 MM charges were used for all residues. Mechanical embedding option was included in the calculations. None of the coordinates were frozen in QM and MM regions.

The geometries of the reactant-complexes (RC), products-complexes (PC), and transition states (TS) were optimized using the 6-31G basis set for all atoms excluding the Zn atom. Single point energies using the 6-31G(d,p) basis set was performed on some selected systems using optimized geometries with the 6-31G basis set. For Zn, the SDD basis set and effective core potential were used.^25,26^ TS structures were validated with one negative eigenvalue, and RC and PC structures were without any negative eigenvalues through frequency calculations. TS structures were validated through intrinsic reaction coordinate (IRC) calculations.^27^ TS structure candidates were first estimated by potential energy surface (PES) scans by scanning the bond coordinates that are forming or breaking, and the maximum energy points in the scans were subjected to TS optimization using Berny algorithm.^28^

**Figure 3:**
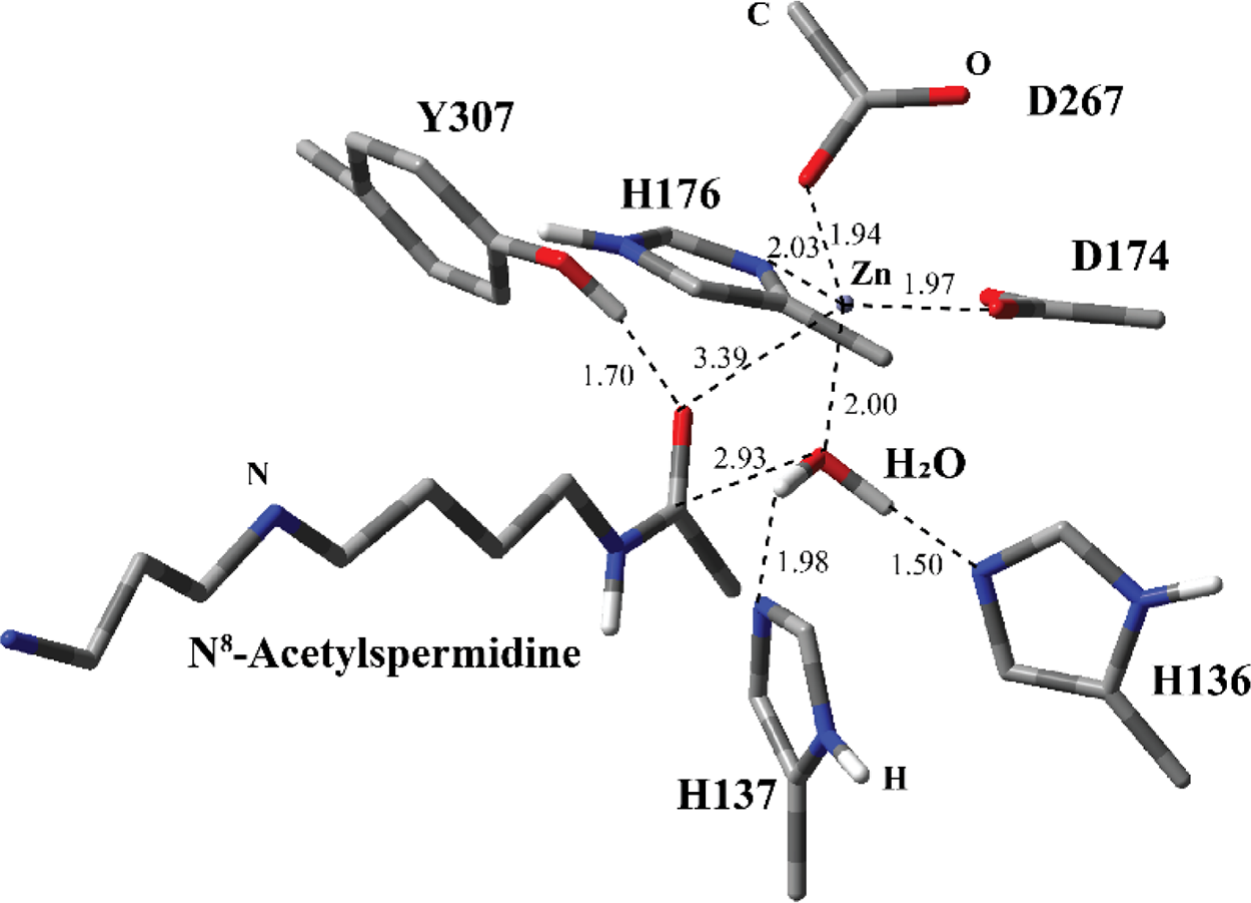
Structure of optimized RC1 including the substrate and catalytically important residues in the QM region belonging to model MS1 obtained with ONIOM(cam-B3LYP/6-31G:Amber) with tube models excluding H atoms except the ones shown with ivory color. The distances are given in Å.

For the ONIOM calculations, a model enzyme–substrate complex was used including N^8^-acetylspermidine and residues around N^8^-acetylspermidine in a radius of 10 Å. This model was generated from the crystal structure of the substrate-bound enzyme (PDB accession code: 7KUQ)^6^ using the VMD program.^29^ F307 is converted to Y307 to produce the native enzyme model. The model has 1208 atoms and 114 residues including Zn^2+^, N^8^-acetylspermidine, and 21 water molecules. Acetyl and *N*-methyl groups were attached to the N-terminal and C-terminal residues on the peripheries. In this way, the electrostatic environment around the active site is not altered. The charges of residues with ionizable groups were determined according to the physiological pH excluding H137. In two different models, H137 was held either neutral or positively charged. The total charge of ONIOM models was either −3 or −4 depending on the protonation state of H137. In the ONIOM model systems, the QM region included Zn^2+^, N^8^-acetylspermidine, an H_2_O molecule, D174, H136, H137, H176, D267, and Y307. The QM region included 100 or 101 atoms together with a total charge of +2 or +3 depending on the protonation state of H137. A part of the QM region residues were included into the MM region, and Figure 3 shows the QM region of these residues. Inclusion of more residues and water molecules into the QM region might portray a better picture of the catalysis. However, due to the cost of computation, only essential residues were included in the QM region.

The initial model structure extracted from the non-productive enzyme–substrate structure (Figure 1a) was subjected to geometry optimization using the cam-B3LYP functional with the 6-31G basis set. With this optimized structure, a PES scan was performed using the distance between the O atom of the active site H_2_O molecule and the carbonyl C atom of the amide group of N^8^-acetylspermidine. The distance was scanned by decreasing the distance in a series of steps to be able to locate the transition state (TS) structure corresponding to the nucleophilic addition of the H_2_O molecule to the carbonyl C of the amide group in N^8^-acetylspermidine. The highest energy point in the scan was employed to locate the optimized TS structure (TS1). Using appropriate points in the PES scan, a reactant complex (RC1), which is the reactive model enzyme–substrate complex, and a product complex (PC1), which is the tetrahedral intermediate, were optimized. IRC calculations were run on the optimized TS to validate these structures. Another PES scan was performed using PC1 structure so as to locate the TS structure (TS2) corresponding to the deacetylation of the tetrahedral intermediate into acetate and spermidine (second step). The PES scan utilized protonation of the N atom of the tetrahedral intermediate by H137. The distance between the H atom at H137 and the N atom at PC1 was scanned by decreasing the distance in a number of steps to be able to locate the TS2, RC2, and PC2.

## 3 Results and Discussions

### 3.1 Nucleophilic H_2_O Addition Step

#### 3.1.1 Reactant Complex (RC1)

In order to probe the role of the protonation states of His dyad in the deacetylation reaction, three model systems were used. According to the proposed mechanism (Figure 2), H136 should be neutral in order to deprotonate the active site H_2_O molecule for the nucleophilic addition of the −OH anion to the carbonyl C atom of N^8^-acetylspermidine. In the first model system (MS1), both H136 and H137 were kept neutral, whereas H137 was positively charged in the second model system (MS2). The optimized geometry of the model productive enzyme–substrate complex (RC1) (Figure 3) for MS1 is very close to the geometry of the non-productive enzyme–substrate complex in the crystal structure (Figure 1a). The first prominent difference between these two geometries is that the carbonyl O atom of N^8^-acetylspermidine has a weaker coordination interaction with Zn^2+^ in RC1 considering almost 1.2 Å more distance. This is presumably as a result of a close H-bonding interaction between Y307 and the carbonyl O atom of N^8^-acetylspermidine, which does not exist in the crystal structure belonging to the Y307F variant. As a result of this interaction, the carbonyl O atom of N^8^-acetylspermidine has less electron density and thereby has weaker coordination interaction with Zn^2+^. The Zn^2+^ ion is coordinated with H176, D174, D267, and a H_2_O molecule, which has H-bonding interactions with the catalytic His dyad consisting of H136 and H137. H136 has stronger H-bonding interaction with the H_2_O molecule than H137 based on its closer distance suggesting that H136 is the catalytic base, which deprotonates the H_2_O molecule during the nucleophilic addition process. H137 is closer to amide N of the acetyl group at N^8^-acetylspermidine with respect to H136 indicating that it might be involved in the protonation of it during the deacetylation step.

#### 3.1.2 Transition State (TS1)

**Figure 4:**
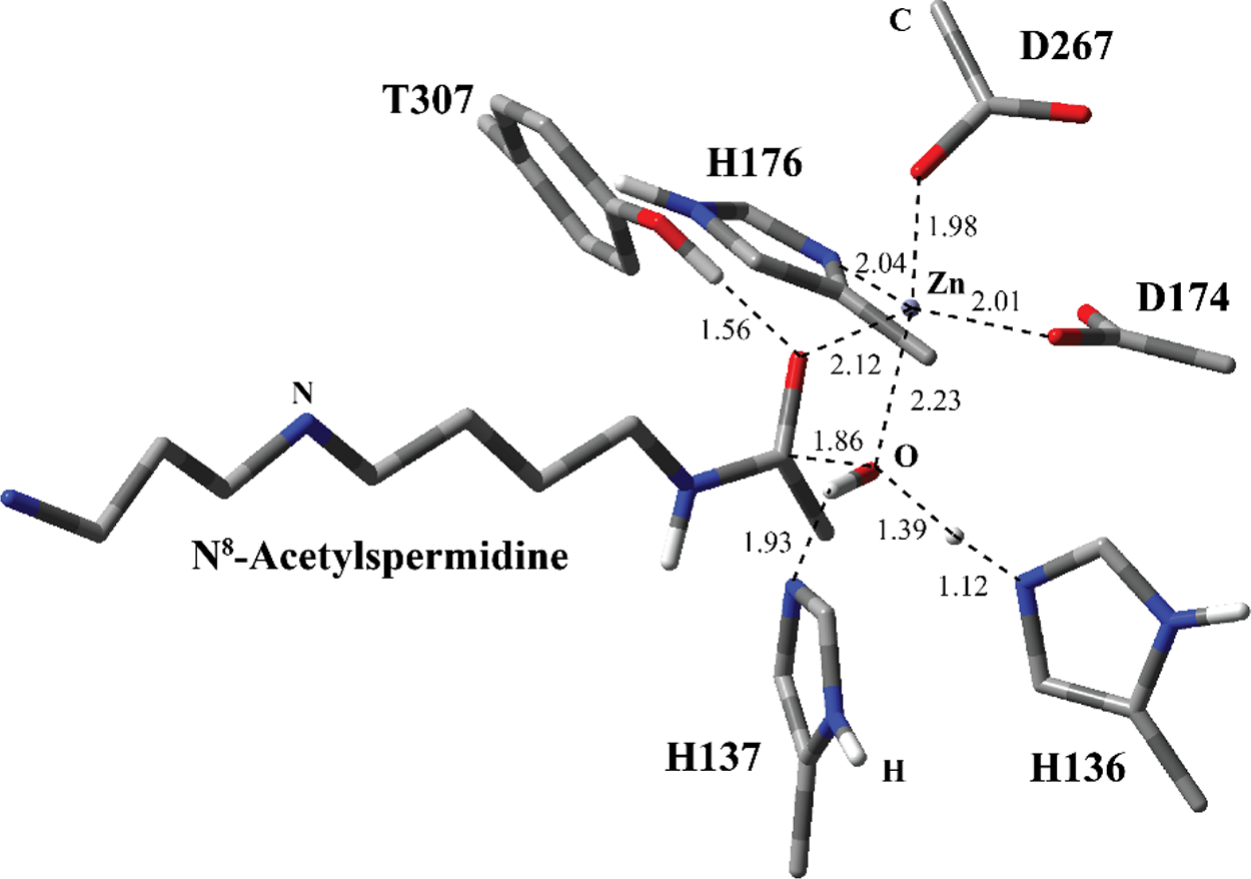
Structure of optimized TS1 including the substrate and catalytically important residues in the QM region belonging to model MS1 obtained with ONIOM(cam-B3LYP/6-31G:Amber) with tube models excluding H atoms except the ones shown with ivory color. The distances are given in Å.

The transition state structure for MS1 (TS1) was obtained from the PES scan using the distance between the O atom at the H_2_O molecule and the carbonyl C atom of N^8^-acetylspermidine. The TS1 structure reflects that as a new bond between the O atom of the H_2_O molecule and the carbonyl C atom of N^8^-acetylspermidine forms, H136 starts deprotonating the H_2_O molecule (Figure 4). In this model, indeed, H136 acts as the catalytic base activating the H_2_O molecule toward nucleophilic addition by deprotonating it. H137 has H-bonding interaction with the O atom of the H_2_O molecule, which is expected to increase the nucleophilicity. As compared to RC1, the carbonyl O atom at the N^8^-acetylspermidine in TS1 has stronger coordination interaction with Zn^2+^ based on 1.70 Å less distance. It is evident that the developing negative charge on the carbonyl O atom during the TS is stabilized by the Zn^2+^. In parallel, the H-bonding interaction of Y307 with carbonyl O atom makes carbonyl C more susceptible to nucleophilic attack. Furthermore, the coordination between the O atom at the H_2_O molecule and Zn^2+^ is weaker based on more distance in TS1 as compared to RC1, which renders the H_2_O molecule more nucleophilic. H176, D174, and D267 are in coordination with the Zn^2+^ ion.

The activation energy as the Gibbs free energy difference between TS1 and RC1 for the MS1 was calculated 15.69 kcal/mol by the cam-B3LYP functional using the 6-31G basis set (Ea-f for the first step at entry #1 in Table 1). The same barrier is estimated to be 21.85 kcal/mol using single point energies with a larger basis set, 6-31G(d,p) with cam-B3LYP. Based on the reported *k_cat_* value of the HDAC 10, which is ca. 0.28 s^–1^,^3^ using Arrhenius equation, an activation energy of ca. 18.2 kcal/mol at 25 °C and 1 atm is expected. Indeed, the calculated activation energies estimated by cam-B3LYP and ωB97XD (20.55 kcal/mol, Ea-f for Step 1 at entry #3 in Table 1) are closer to this value than that of the M06-2X functional (25.59 kcal/mol, Ea-f for step 1 at entry #2). This finding indicates that ωB97XD and cam-B3LYP provide complementary computational results to experimental studies for HDAC 10. However, ωB97XD and M06-2X functionals produced RC1 structures (Cartesian coordinates of the QM regions are provided in the Supporting Information) that did not show the H-bonding interaction between Y307 and carbonyl O of the substrate. For that reason, cam-B3LYP functional depicts the nucleophilic addition step more properly as compared to the two other functionals. The TS structures were estimated similarly by three functionals.

**Table 1:** Energy Profile for the HDAC10 for the Model System 1 (MS1, First Step) and Model System 3 (MS3, Second Step) Using Cam-B3LYP, M06-2X, ωB97XD Functionals in the QM Region (Ea-f: Activation Energy for the Forward Reaction in kcal/mol, Ea-r: Activation Energy for the Reverse Reaction in kcal/mol; The Values in Parentheses Belong to Activation Energies in Terms of Absolute Energy Difference Calculated with Single Point Energies Using 6-31G(d,p) Basis Set on; All the Other Activation Energies Are Based on Gibbs Free Energy Change with the 6-31G Basis Set)

		first step		second step		imaginary frequency (*i*)	
entry #	functional	Ea-f	Ea-b	Ea-f	Ea-r	first step	second step
1	cam-B3LYP	15.69 (21.85)	3.19 (4.49)	1.37 (2.18)	22.28 (28.33)	–270.73	–154.84
2	M06-2X	25.59	3.60	2.37	20.94	–311.83	–178.93
3	ωB97XD	20.55	2.95	1.16	22.72	–265.96	–177.76

The activation energy (Ea) as the Gibbs free energy difference between RC1 (Figure S1) and TS1 (Figure S2) for the second model, MS2, in which H137 was positively charged was calculated to be 109.49 kcal/mol with the 6-31G basis set with the cam-B3LYP functional. This is an unusually high activation barrier. The same energy barrier was calculated to be 45.73 kcal/mol by M06-2X and 54.41 kcal/mol by ωB97XD. These values are still considerable barriers. A careful analysis of the TS1 (Figure S2) structure for MS2 with the cam-B3LYP functional reveals several clues for this unusual barrier. First, a steric interaction between positively charged H137 and the H_2_O molecule pushes the O atom at H_2_O to be closer to Zn^2+^, which decreases the nucleophilicity. Second, the H-bonding interaction between the N atom in neutral H137 and the H atom of the H_2_O molecule is lost upon the protonation of the N atom in H137, which further decreases the nucleophilicity of H_2_O. These electronic as well as steric factors may have caused an unfavorable activation barrier. A similar high activation barrier was reported for the HDAC 8 enzyme using ab initio QM/MM MD simulations with umbrella sampling.^11^ In the same manner, QM-MM calculations for the histone-deacetylase-like protein (HDLP) also supported singly protonated His residues for the catalytic His dyad.^14^ Based on our results and also reported systems, H137 is likely to be neutral during the nucleophilic addition step. Furthermore, the optimized RC1 structure for MS2 (Figure S1) with cam-B3LYP corresponded to positively charged H136 and neutral H137. It indicates that H137 cannot stay positively charged as a result of proton transfer to H136 relayed by the H_2_O molecule. This also suggests that H136 is more basic than H137.

For HDAC 8, the analog of the H137 was proposed to be both acting as the general base, which deprotonates the active site H_2_O molecule, and as the general acid, which subsequently protonates the amide N of the substrate.^30,31^ This was supported by experimental^32^ and computational works.^11^ In our study, the relaxed PES scan between the O atom of H_2_O molecule and the carbonyl C of the substrate indicated that H136 acts as the catalytic base. Furthermore, we conducted another PES scan between the H atom of the H_2_O molecule and the N atom of H137 to investigate the possibility of the H137 as the catalytic base. The PES scan generated an uphill energy profile implying that H137 cannot act as the catalytic base to deprotonate the H_2_O molecule. The lack of basicity of H137 in HDAC 10 was attributed to the H-bonding interaction of H137 with glutamine, which does not raise its pKa with respect to the H-bonding interaction of H137 with aspartate in HDAC 8.^6^

#### 3.1.3 Product Complex (PC1)

**Figure 5:**
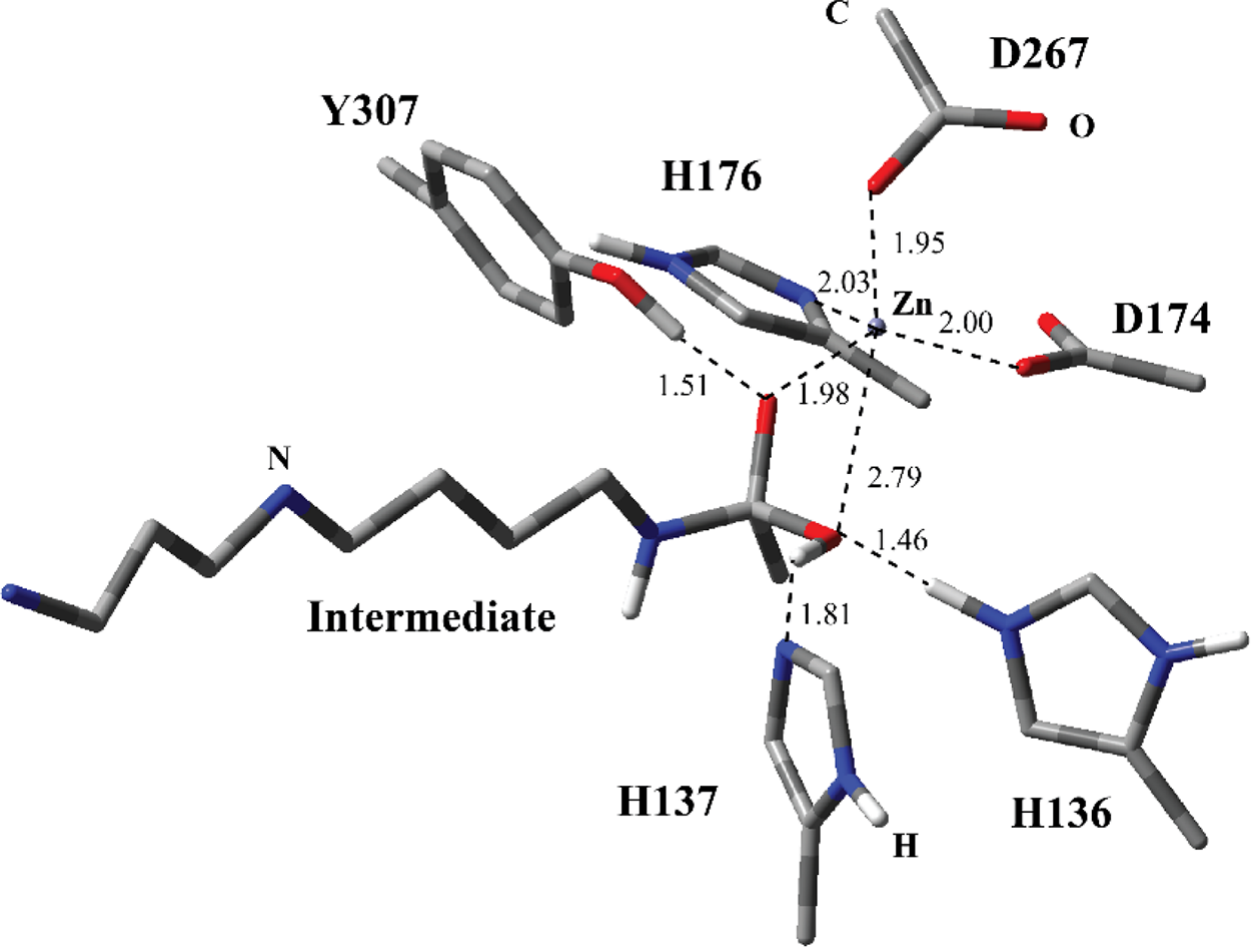
Structure of optimized PC1 including the substrate and catalytically important residues in the QM region belonging to MS1 obtained with ONIOM(cam-B3LYP/6-31G:Amber) with tube models excluding H atoms except the ones shown with ivory color. The distances are given in Å.

The PC1 for the nucleophilic addition step for the MS1 was optimized using a downhill energy point in the PES scan (Figure 5). The C–OH bond formed between carbonyl C of the N^8^-acetylspermidine and O of the H_2_O molecule while H136 became protonated. This intermediate adduct structurally resembles to the TS1 structure (Figure 4) and has similar H-bonding distances and coordination distances for Zn^2+^. The O atom of OH (previously H_2_O molecule) loosely coordinates the Zn^2+^ and the O atom of the oxyanion (previously carbonyl group) has stronger coordination interaction. Based on the structural similarities of the TS1 (Figure 4) and PC1 structures (Figure 5), it could be inferred that the nucleophilic addition of H_2_O is a highly endergonic process. Indeed, the calculated activation barrier for the reverse process turned out to be 3.19 kcal/mol (Ea-r for step 1 for entry #1 in Table 1) with cam-B3LYP with the 6-31G basis set. The other two functionals also estimated a small activation barrier for the reverse process. The intermediate adduct oxyanion is stabilized by H-bonding interaction with Y307 and coordination with the catalytic Zn^2+^. According to the proposed mechanism, the amide N of this reactive intermediate will be protonated by H137 and the acetyl group will be eliminated. Herbst-Gervasoni and Christianson^6^ was able to report the crystal structure of this reactive adduct using the H137A variant (Figure 1b). This gives us a chance to compare the structure of the experimentally obtained intermediate adduct to the computationally obtained one. The cam-B3LYP DFT functional produced a very similar adduct structure (Figure 5) with respect to the crystal structure (Figure 1b). The calculated distances for the Zn^2+^ coordination are very close to the crystal structure values.

### 3.2 Deacetylation Step

Based on the proposed mechanism (Figure 2), following the nucleophilic H_2_O addition step, the deacetylation of the intermediate adduct occurs as a result of protonation of the amide N by H137. However, as stated previously, the calculated activation barrier is unusually high if H137 is positively charged during the H_2_O addition step. This observation suggests that either H137 gains a proton following the H_2_O addition step, or another catalytic acid should protonate the amide N for the deaceylation step. Herbst-Gervasoni and Christianson^6^ showed that when H137 was mutated to H137A, the crystal structure of the enzyme-tetrahedral intermediate adduct was obtained. This clearly indicates the important role of H137 for the deacetylation step. A careful analysis of the PC structure (Figure 5) shows that in the MM region, there are two H_2_O molecules which are 4.0–5.0 A° away from H137. One of these H_2_O molecules might diffuse in the form of a hydronium ion and protonate H137. The protonation of H137 deserves further detailed computational studies. As future studies, model systems including extra water molecules in the vicinity might be employed to study the protonation mechanism of H137.

In order to build a model system for the deacetylation step (model system 3, MS3), the product of the H_2_O addition step (Figure S3) for the model system (MS2) in which H137 was positively charged was used to locate the reactant complex (RC2), transition state (TS2), and product complex (PC2) through PES scans. The distance between the amide N and carbon was increased over a number of steps to model the deamination/deacetylation process. The highest energy point was used to optimize the TS2, and proper downhill points were used to optimize the RC2 and PC2 structures for MS3.

#### 3.2.1 Reactant Complex (RC2)

**Figure 6:**
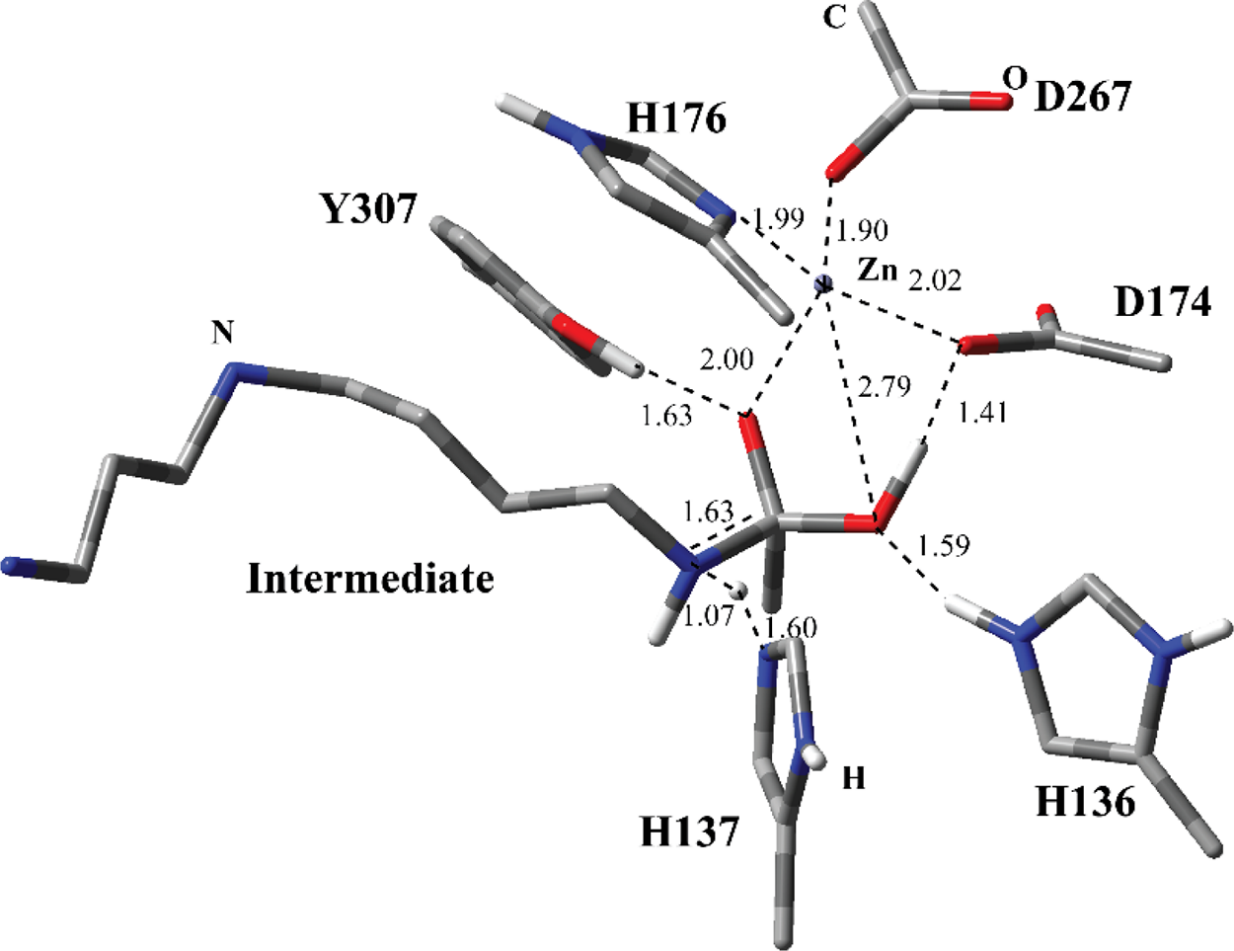
Structure of optimized RC2 including the substrate and catalytically important residues in the QM region belonging to MS3 obtained with ONIOM(cam-B3LYP/6-31G:Amber) with tube models excluding H atoms except the ones shown with ivory color. The distances are given in Å.

The optimized geometry of the RC2 (Figure 6) for MS3 reveals important clues for the deacetylation step. The first striking feature is that the adduct’s N atom seems already protonated by H137. It could be concluded that the protonation of the N atom by H137 is a downhill process when H137 is positively charged and doubly protonated. In addition, the C–N bond (1.63 A°) is longer than that of PC1 (1.43 A° in Figure 5), suggesting that the protonation of the N atom in the adduct weakens the C–N bond. Another prominent feature in the RC2 structure is the new H-bonding interaction of the adduct with D174. Presumably, the protonation of the N atom in the adduct sterically forced the H atom at the OH group to rotate and have H-bonding with D174. Furthermore, the conformation of the adduct changed from a straight chain to bent. This might have been caused by the protonation of the N atom by H137, which may subsequently change the interaction of the adduct with the surrounding residues. The coordination environment of the Zn^2+^ is similar to the one in the PC1 (Figure 5). Zn^2+^ is tetra coordinated by the oxyanion of the adduct, H176, D174, and D267. The oxyanion is further stabilized by the H-bonding interaction with Y307.

#### 3.2.2 Transition State (TS2) and Product Complex (PC2)

**Figure 7:**
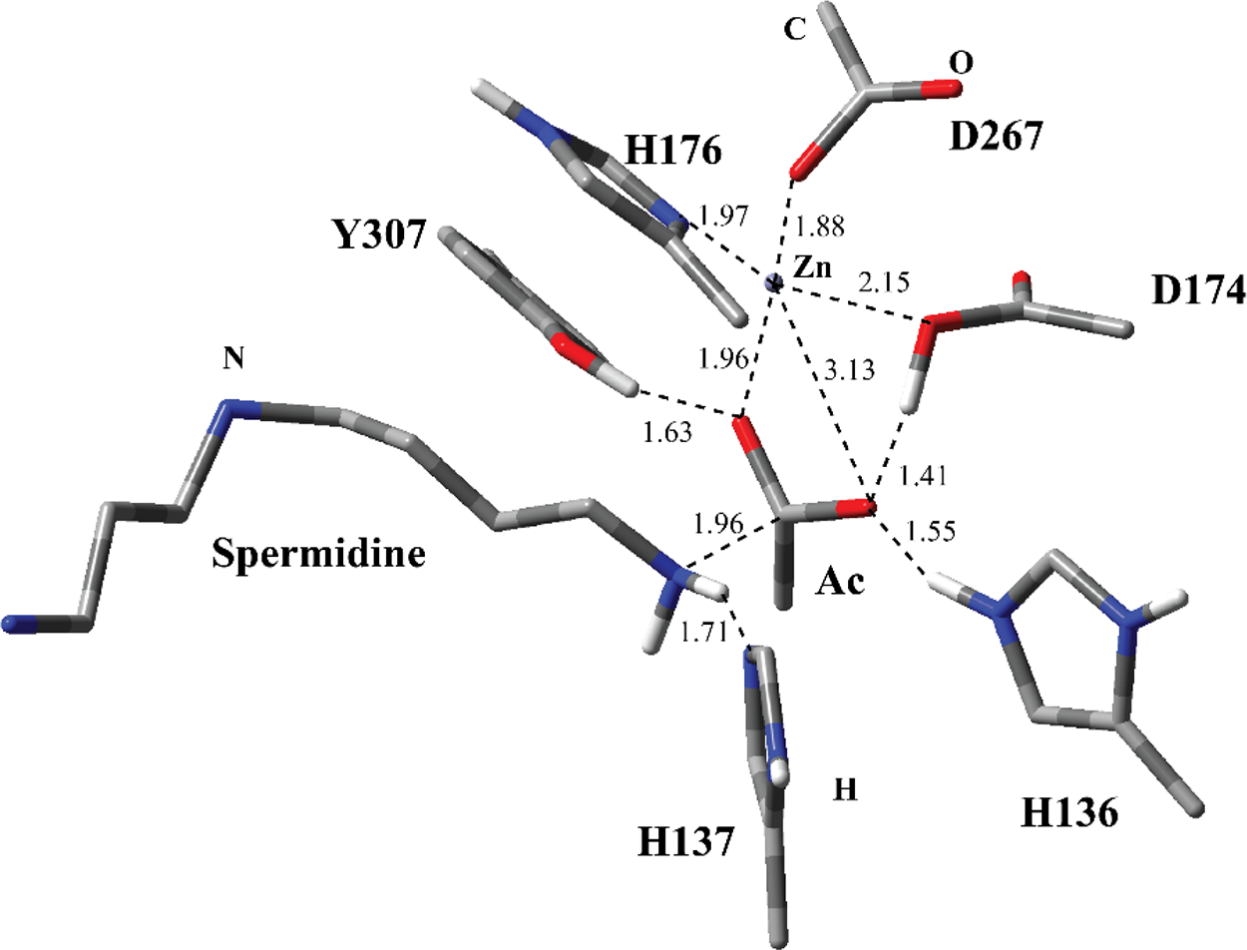
Structure of optimized TS2 including the substrate and catalytically important residues in the QM region belonging to MS3 obtained with ONIOM(cam-B3LYP/6-31G:Amber) with tube models excluding H atoms except the ones shown with ivory color. The distances are given in Å.

The optimized geometry of the TS2 structure (Figure 7) for MS3 shows similar interactions and structurally similar to RC2 (Figure 6). The C–N bond in the intermediate is separated further resulting in spermidine and acetate ions. The acetate ion (Ac in Figure 7) has close H-bonding interactions with Y307 and D174. In essence, the proton is transferred from acetate to D174. Zn^2+^ is coordinated by the acetate anion, H176, D174, and D267. Similarly, H137 and spermidine has close H-bonding interaction. The proton is completely transferred to spermidine’s N from H137.

**Figure 8:**
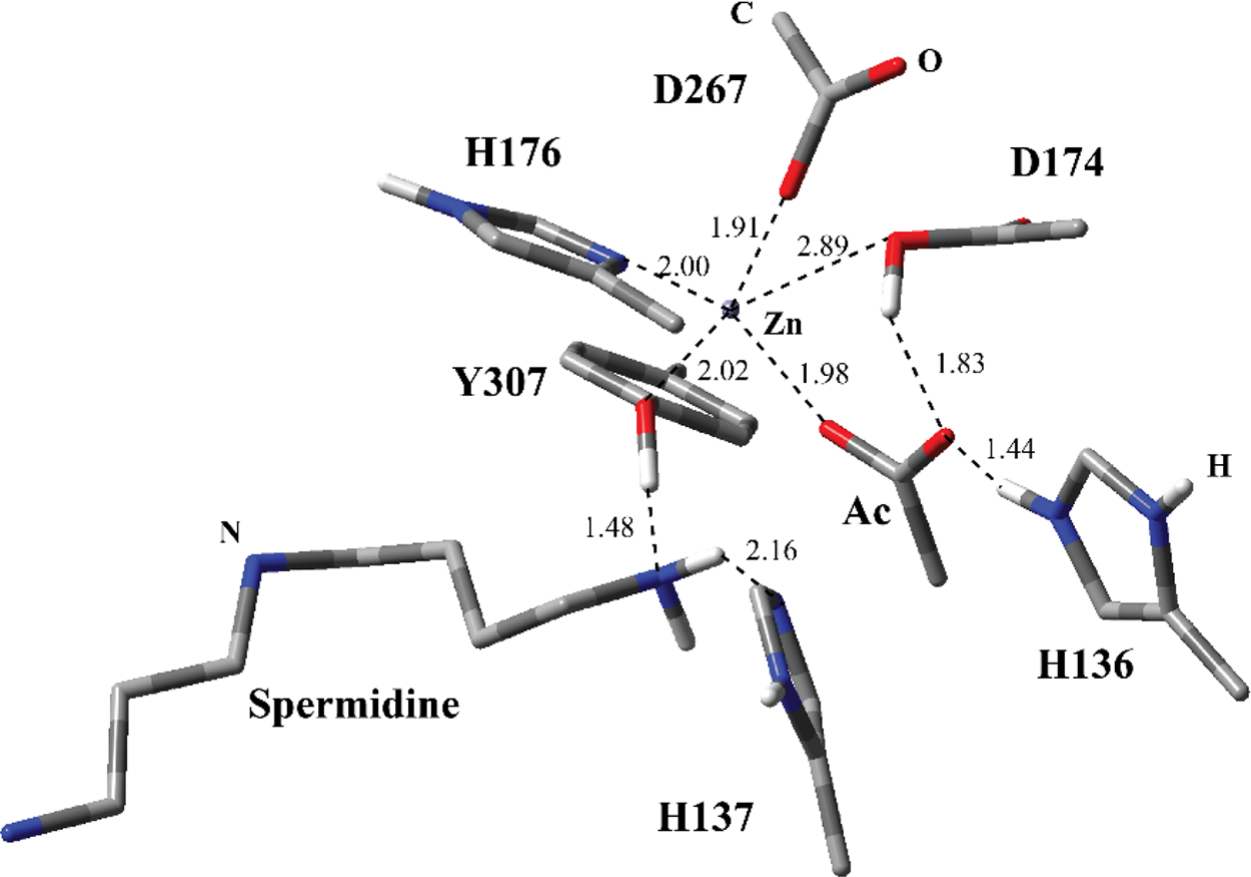
Structure of optimized PC2 including the substrate and catalytically important residues in the QM region belonging to MS3 obtained with ONIOM(M06-2X/6-31G:Amber) with tube models excluding H atoms except the ones shown with ivory color. The distances are given in Å.

The energy barrier for the deacetylation step is predicted to be small, and it is a very exergonic process. (Ea-f for the second step in Table 1) All the three functionals estimated similar barriers around 1–2 kcal/mol. In parallel, all three functionals produced similar RC2 and TS2. However, the PC2 structures did not converge for cam-B3LYP and ωB97XD functionals. For that reason, single point energies of the PC2 structure, optimized with M06-2X, were calculated with these two functionals to calculate the activation barrier of the reverse step (Ea-r for the second step in Table 1) as the absolute energy difference between TS2 and PC2. The optimized PC2 geometry for the deacetylation step obtained with the M06-2X functional (Figure 8) shows that the intermediate adduct is broken down into acetyl and spermidine parts completely. Y307 has H-bonding interactions with spermidine instead of the acetate part. The acetate part has H-bonding interactions with H136 and D174 in addition to the coordination interaction with Zn^2+^. Similar to TS2 and RC2, Zn^2+^ is coordinated with H176, D267, and D174. Based on the PC2 structure, spermidine moved away from the active site. A very exergonic energy profile with a reverse activation barrier of 20.94 kcal/mol with M06-2X functional (Ea-r for the second step at entry #2 in Table 1) implies that the deacetylation products are more stable than the intermediate adduct. Similar values were obtained as absolute energy differences for cam-B3LYP and ωB97XD functionals.

## 4 Conclusions

In this study, we investigated the deacetylation mechanism of HDAC 10 with ONIOM calculations. The AMBER force field and three different DFT functionals were used to analyze nucleophilic H_2_O addition and deacetylation steps in model systems. It was found that H136 acts as the catalytic base deprotonating the H_2_O molecule in a rate determining step. Furthermore, it is also found that during the H_2_O addition step, H137 is required to be neutral. The second step, deacetylation process, requires H137 to be positively charged suggesting that it has to be protonated following the H_2_O addition step. Our models highlighted the structure of the active site during these steps and the potential roles of residues and Zn^2+^ ion in the enzymatic transformation. cam-B3LYP and ωB97XD functionals estimated reasonable activation barriers based on the reported *kcat* value. Further studies are required to understand the protonation of H137 following the H_2_O addition step. Our study provides invaluable insights in investigating and understanding the mechanism of HDAC 10.

## Supporting Information Available

Figures associated with model systems (Figures S1–S3); tables (Tables S1–S3) associated with Gibbs free energy, enthalpy, zero-point corrected electronic energies of ONIOM calculations with 6-31G basis set with three functional; and Cartesian coordinates of all species in three model systems (PDF)
