# Supplementary material for: Computational Analysis of Histone Deacetylase 10 Mechanism by the ONIOM Method: A Complementary Approach to X-ray and Kinetics Studies
Source: ACS Omega. 2022 Feb 9;7(7):6393–402. doi: 10.1021/acsomega.1c07055 (PMC8868106; doi:10.1021/acsomega.1c07055)
Supplement: Supplementary file 1 — ao1c07055_si_001.pdf [file ao1c07055_si_001.pdf]

Computational Analysis of Histone Deacetylase 10 Mechanism by ONIOM Method: A Complementary Approach to X-ray and Kinetics Studies

Author(s): Ibrahim Yildiz[†*], Banu Sizirici Yildiz[‡]

[†]Khalifa University, Chemistry Department, PO Box 127788, Abu Dhabi, UAE Tel: +971 (0)2 401 8208

[‡]Khalifa University, CIVE Department, PO Box 127788, Abu Dhabi, UAE

*E-mail: ibrahim.yildiz@ku.ac.ae

Supporting Figures

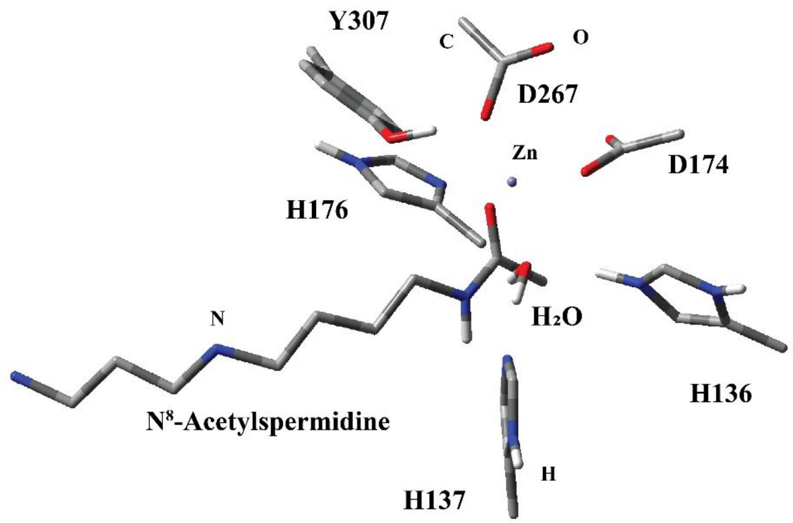

Figure S1. The structure of optimized RC1 for MS2 including substrate and catalytically important residues in QM region obtained with ONIOM(cam-B3LYP/6-31G:Amber) tube models excluding H atoms except the ones shown with ivory color.

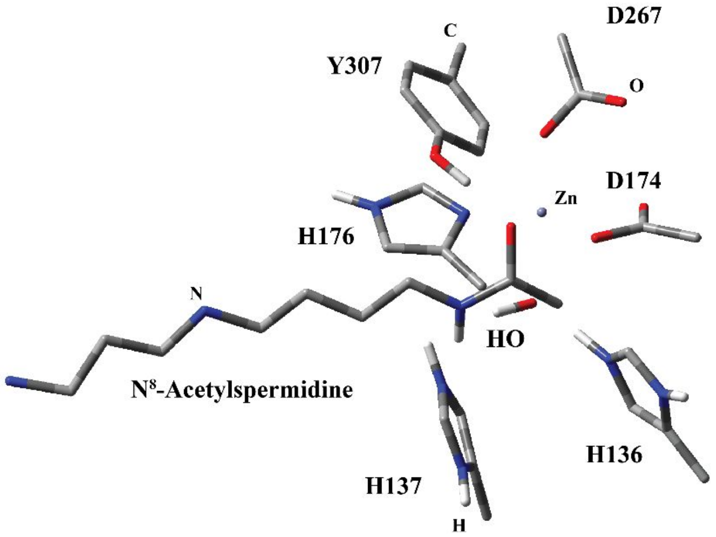

Figure S2. The structure of optimized TS1 for MS2 including substrate and catalytically important residues in QM region obtained with ONIOM(cam-B3LYP/6-31G:Amber) tube models excluding H atoms except the ones shown with ivory color.

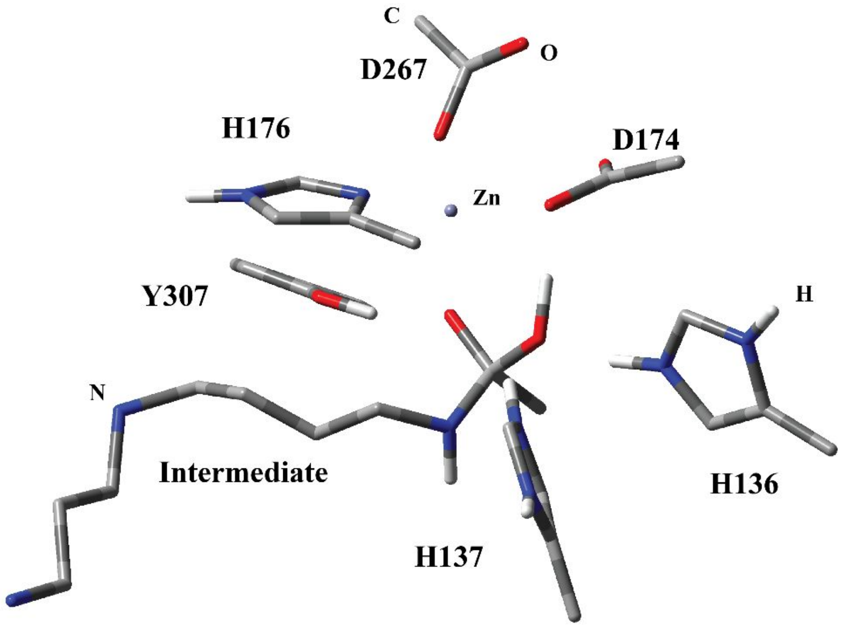

Figure S3. The structure of optimized PC1 for MS2 including substrate and catalytically important residues in QM region obtained with ONIOM(cam-B3LYP/6-31G:Amber) tube models excluding H atoms except the ones shown with ivory color.

Supporting Tables

Table S1 Absolute Energies of Reactant Complex, Product Complex, and Transition State for the Hydride Transfer Process for MS1 (Entry #1 in Table 1) with cam-B3LYP functional with 6-31G basis set (ZPE: Zero-point energy correction, H: Enthalpy, G: Gibbs free energy)

**Species**	**E+ZPE (au)**	**H (au)**	**G (au)**	**Imaginary Frequency (*i*)**
**RC1**	-2492.71	-2492.04	-2493.45	
**TS1**	-2492.68	-2492.02	-2493.43	-270.73
**PC1**	-2492.69	-2492.02	-2493.43	

Table S2. Absolute Energies of Reactant Complex, Product Complex, and Transition State for the Hydride Transfer Process for MS2 (Figure S1-3) with cam-B3LYP functional with 6-31G basis set (ZPE: Zero-point energy correction, H: Enthalpy, G: Gibbs free energy)

Species	E+ZPE (au)	H (au)	G (au)	Imaginary Frequency (*i*)
**RC1**	-2493.22	-2492.56	-2493.97	
**TS2**	-2493.06	-2492.39	-2493.80	-256.10
**PC2**	-2493.13	-2492.46	-2493.87	

Table S3. Absolute Energies of Reactant Complex, Product Complex, and Transition State for the Hydride Transfer Process for MS3 (2nd Step at Entry #2 in Table 1) with M02-6X functional with 6-31G basis set (ZPE: Zero-point energy correction, H: Enthalpy, G: Gibbs free energy)

Species	E+ZPE (au)	H (au)	G (au)	Imaginary Frequency (*i*)
**RC1**	-2493.47	-2492.81	-2494.21	
**TS2**	-2493.46	-2492.80	-2494.21	-178.93
**PC2**	-2493.50	-2492.84	-2494.24	

Cartesian Coordinates of QM Regions of the Optimized Geometries of All Species with ONIOM Models

MS1 with cam-B3LYP

<u>RC1</u>

C -3.13546922 3.29755132 0.11903988

H -4.10616722 3.25225732 0.62075088

H -2.90793522 4.36171032 0.03872788

C -3.22874722 2.70325932 -1.24466112

N -4.37453322 2.86249132 -2.01808712

C -4.17310222 2.31017232 -3.24202412

H -4.86197322 2.32981732 -4.07184612

N -2.94885122 1.80775932 -3.29964012

C -2.35812622 2.03455432 -2.06531012

H -1.34711422 1.73593632 -1.87370512

C -1.38276522 -1.98184268 -0.28507612

H	-0.36477422	-1.68809168	-0.53749412
H	-1.33544122	-3.03608368	0.01853888
C	-2.18909422	-1.88068168	-1.53128212
N	-3.51256022	-2.27560768	-1.66701712
C	-3.86259022	-2.17064368	-2.98578512
H	-4.84650522	-2.40738468	-3.34995312
N	-2.83723022	-1.73918468	-3.70524212
H	-2.56985222	1.11069132	-4.56714112
C	-1.79443422	-1.55328768	-2.80249612
H	-0.83639122	-1.18402368	-3.12411912
C	-4.68491822	4.82937332	-5.43755412
H	-3.96839422	5.61534232	-5.19226112
H	-5.11464622	4.44176332	-4.50950212
C	-3.95098722	3.73983932	-6.17526412
O	-2.86876922	3.97434732	-6.74710712
O	-4.53525922	2.54507132	-6.20103712
C	-5.78013322	-0.68961368	-5.73041812
H	-5.09100722	0.03265632	-5.28519312
H	-5.91283322	-1.48947368	-4.99732812
C	-5.12096822	-1.26229768	-6.93490912
N	-4.18689222	-0.51634068	-7.65547612
C	-3.63554822	-1.30917468	-8.56695712
H	-2.83254322	-1.04419768	-9.24094412
N	-4.19099722	-2.54643768	-8.46898312
H	-3.88120922	-3.36191168	-9.00168012
C	-5.13638622	-2.53084968	-7.44564412
H	-5.67093722	-3.41653468	-7.15671512
C	-3.07879022	2.84446532	-11.04644412

H	-3.59017222	1.97869832	-11.47357412
H	-2.01401522	2.61663732	-11.12019612
C	-3.46133322	2.92738832	-9.57774612
O	-4.15385322	3.85328332	-9.12027912
O	-2.99611322	1.88369232	-8.89196612
C	3.96449878	0.41202532	-12.70270412
H	4.39659178	-0.47933868	-13.17420412
H	4.78408978	0.88991132	-12.15498212
C	2.86652178	0.05024932	-11.73313012
C	2.84626578	0.59500832	-10.44473712
H	3.70860678	1.15059432	-10.09339212
C	1.74452878	0.45079532	-9.60777412
H	1.75335278	0.86212432	-8.60847612
C	0.62134978	-0.24759468	-10.05996212
O	-0.53593822	-0.36578368	-9.31450312
H	-0.51089222	0.15578432	-8.47374012
C	0.65177678	-0.84881268	-11.30988712
H	-0.22574922	-1.37965168	-11.65547512
C	1.76356378	-0.71131068	-12.12874012
H	1.72816678	-1.16536568	-13.11350012
N	-0.63786522	-11.81726768	-6.58491112
C	0.39057278	0.44701032	-5.94626912
O	0.01520378	0.91124232	-7.04508112
N	-0.68617322	-6.81308368	-7.17260912
N	0.25998678	-0.86944168	-5.65659212
C	-0.13090122	-8.06040468	-6.56169612
C	-0.31149222	-10.42920168	-6.09081612
C	-0.10095222	-5.57914868	-6.48737112

C	-0.42194622	-1.80252368	-6.53517312
C	-0.79283022	-9.34406668	-7.04476512
C	-0.75364122	-4.28811368	-6.91848112
C	-0.23009322	-3.20052468	-5.98047912
C	1.01032678	1.30719032	-4.89431812
Zn	-3.35498922	1.24911032	-7.09555212
O	-2.49308022	0.52012432	-5.44478912
H	-2.63438422	-0.41567368	-5.15768812
H	-0.22057622	-12.50275468	-5.93883912
H	-0.14806022	-11.96201468	-7.49286112
H	-0.47424222	-6.77905468	-8.18519612
H	0.60921178	-1.22971068	-4.77169812
H	0.93431478	-8.06261068	-6.79903912
H	-0.25159622	-7.94449568	-5.48368712
H	0.77759278	-10.40858568	-5.99547612
H	-0.74878722	-10.32020868	-5.09873512
H	-0.23157322	-5.75711968	-5.42218612
H	0.95968178	-5.59574868	-6.73019712
H	-1.48641222	-1.55863068	-6.59888812
H	-0.01972722	-1.73169768	-7.54856312
H	-0.49664522	-9.56152468	-8.07798012
H	-1.88494422	-9.26267468	-7.01735412
H	-0.48798922	-4.05087168	-7.95414912
H	-1.84822522	-4.35251368	-6.85573312
H	-0.72849522	-3.28765868	-5.00839612
H	0.84387878	-3.36191468	-5.82585912
H	1.59936478	0.71026732	-4.19100612
H	0.21830378	1.81514932	-4.33477212

H	1.64410678	2.05955032	-5.35480012
H	-1.63261222	-11.98632368	-6.71455012
H	-1.71500522	-6.78362268	-7.11256712
H	-4.12844922	-2.57015968	-0.91552312
H	-5.24617922	3.32235132	-1.74604912

TS1 (Transition State)

C	-1.45097976	0.48895898	0.01899436
H	-2.43022176	0.46082098	0.50194836
H	-1.21915076	1.54943198	-0.09008164
C	-1.50862176	-0.14256002	-1.32823264
N	-2.61677376	0.01221698	-2.16393564
C	-2.38544176	-0.57545002	-3.35545764
H	-2.99944176	-0.58145702	-4.24654164
N	-1.17005076	-1.10003002	-3.31578264
C	-0.61300176	-0.85364702	-2.07532664
H	0.38495724	-1.15869802	-1.84263864
C	0.24913124	-4.82930302	-0.14834664
H	1.27378224	-4.55524902	-0.39399864
H	0.27951224	-5.87082802	0.19557936
C	-0.53631476	-4.76609402	-1.40762564
N	-1.87109276	-5.11960602	-1.54129364
C	-2.20379776	-5.05976102	-2.86724964
H	-3.19189476	-5.27504502	-3.23293364
N	-1.15709476	-4.69609902	-3.59035464
H	-0.75115076	-1.68703402	-4.17334764
C	-0.11615076	-4.51016602	-2.68675864
H	0.86166324	-4.20247702	-3.01642664

C	-2.78952676	1.83409098	-5.52965464
H	-2.07898576	2.60007198	-5.21263464
H	-3.27467576	1.41605998	-4.64393264
C	-2.01083676	0.78735698	-6.29839364
O	-1.00752676	1.13099998	-6.94713564
O	-2.46131676	-0.46492102	-6.25188464
C	-3.81962476	-3.52976602	-5.76705664
H	-3.14170276	-2.76258502	-5.38787764
H	-3.92713876	-4.28373102	-4.98232364
C	-3.18144076	-4.16275702	-6.95171064
N	-2.21098176	-3.48176302	-7.68831364
C	-1.74530276	-4.30996102	-8.61631964
H	-0.94008376	-4.11070302	-9.31046164
N	-2.38328676	-5.50529302	-8.50751464
H	-2.12975276	-6.33871402	-9.04213064
C	-3.29911276	-5.42721502	-7.46093564
H	-3.89349076	-6.27136202	-7.16412264
C	-1.34624476	-0.11562602	-11.21485464
H	-1.96042476	-0.91314302	-11.64068364
H	-0.31249676	-0.45680502	-11.30518564
C	-1.68918076	0.00022898	-9.73424064
O	-2.34386476	0.95991498	-9.27977464
O	-1.25029376	-1.03975302	-9.04754264
C	5.88271324	-2.50900002	-12.39572864
H	6.43457824	-3.37340002	-12.78666764
H	6.59749324	-1.94588102	-11.78758064
C	4.72552124	-2.93615202	-11.52613864
C	4.55099124	-2.40203802	-10.24375364

H	5.33512024	-1.78846402	-9.81363264
C	3.39281024	-2.63997002	-9.50902964
H	3.28203624	-2.26484302	-8.49966964
C	2.36448724	-3.40423402	-10.06511764
O	1.16548824	-3.60334902	-9.41919464
H	1.04220524	-3.04667702	-8.58846964
C	2.54987524	-3.99052302	-11.30916664
H	1.74640024	-4.57364302	-11.73812164
C	3.72084024	-3.77262802	-12.01895464
H	3.81172924	-4.22926502	-12.99776964
N	0.99892024	-15.02158902	-6.53546264
C	1.22020624	-2.64656002	-6.07011164
O	0.82727924	-2.24793802	-7.26227564
N	1.05019624	-10.02992902	-7.16630464
N	1.57536024	-3.97051302	-5.90500264
C	1.50808824	-11.27995702	-6.49197464
C	1.21526724	-13.63057402	-5.98962464
C	1.67135024	-8.79532902	-6.51003864
C	0.93594924	-5.04399902	-6.64383864
C	0.82850324	-12.54782502	-6.99010964
C	1.01598124	-7.52808702	-6.99671364
C	1.48656924	-6.36283102	-6.13671964
C	2.02724924	-1.70488302	-5.21880664
Zn	-1.24106776	-1.75341202	-7.20507864
O	-0.45233176	-2.50463102	-5.26317364
H	-0.74613276	-3.40656302	-4.96997764
H	1.40047624	-15.70307502	-5.87569764
H	1.56580224	-15.11743402	-7.40450764

H	1.29809324	-10.03357602	-8.17140464
H	2.05118924	-4.24074702	-5.04563164
H	2.58580924	-11.33470702	-6.65443664
H	1.31997124	-11.13032702	-5.42739764
H	2.28374024	-13.58121602	-5.76225264
H	0.65741624	-13.54451102	-5.05779864
H	1.53768024	-8.93799402	-5.43962964
H	2.73063324	-8.83615002	-6.75717564
H	-0.15200776	-5.03313702	-6.49669564
H	1.13179624	-4.93360902	-7.71382764
H	1.18090924	-12.79332502	-7.99948464
H	-0.25923776	-12.42828702	-7.03448164
H	1.27964224	-7.33748002	-8.04211164
H	-0.07883676	-7.61189702	-6.93296564
H	1.16446924	-6.51980202	-5.10050864
H	2.57991524	-6.31872902	-6.15007064
H	2.19752624	-2.13517802	-4.22531864
H	1.51213124	-0.76146402	-5.11003764
H	2.99709024	-1.53481202	-5.69045664
H	0.02947324	-15.23869802	-6.75411764
H	0.02322324	-9.93859702	-7.14353764
H	-2.49786976	-5.38528102	-0.78861564
H	-3.48143276	0.51529198	-1.95046664

PC1 (Product Complex)

C	4.44146200	2.87594000	-2.18715000
C	5.22116900	2.74809000	-0.91765600
C	6.53349000	3.04789600	-0.68750300

C	4.70212800	2.25497400	0.33053800
N	6.86835300	2.75764100	0.62500500
C	5.75688800	2.28150500	1.27521400
C	3.43082600	1.81266300	0.72466100
C	5.57235300	1.87912300	2.60517200
C	3.24543800	1.41510100	2.03839000
C	4.30528300	1.44637000	2.96908800
C	0.93935400	-4.44737300	-0.30793100
C	-0.26645100	-3.51748000	-0.20565600
C	0.84893300	-5.62042200	0.66489500
C	-3.49554500	-4.78208000	-2.21104100
C	-3.06111500	-3.46065300	-2.78818200
C	-3.96785000	-2.41891500	-2.98265200
C	-1.74021600	-3.26869700	-3.20755300
C	-3.58370700	-1.23775400	-3.61660800
C	-1.33604700	-2.09138400	-3.82138600
C	-2.26866600	-1.07877700	-4.04484000
O	-1.83182000	0.05073100	-4.68652800
C	4.45598800	-4.76853500	1.03905800
C	4.90928700	-3.36000100	0.81411000
C	4.63998900	-2.26438200	1.59348500
C	5.73318800	-2.88514500	-0.26797700
N	5.23478000	-1.14515900	1.06279300
C	5.91027800	-1.49117200	-0.07801200
C	6.30723500	-3.49378700	-1.39670100
C	6.62044400	-0.69373200	-0.98243000
C	7.01822600	-2.71251500	-2.29006000
C	7.17004800	-1.32532100	-2.08446900

C	1.46718500	-2.63507900	2.97206600
O	1.80121000	-1.25842600	2.95370400
C	0.05316400	-2.82939800	3.49156500
C	-7.05488200	0.35891000	0.07123800
C	-6.52765000	-1.04088100	0.03220300
C	-7.03741300	-2.10330500	-0.65870800
C	-5.38843000	-1.54380300	0.75931200
N	-6.27774800	-3.23525200	-0.42214700
C	-5.26177200	-2.91832600	0.45153500
C	-4.48731700	-0.95910000	1.66263000
C	-4.25035800	-3.71389700	1.00086800
C	-3.48581900	-1.74266700	2.21148200
C	-3.36583000	-3.10576400	1.87647000
N	1.90636600	-1.03030000	0.14440000
C	0.74342900	-0.40522800	0.01581700
C	0.27093200	0.10624600	-1.20080700
C	1.06638100	-0.06832100	-2.33560200
C	2.28287200	-0.72748700	-2.20249400
C	2.66286900	-1.19453800	-0.94826300
N	-1.49372800	1.59616600	-2.05606100
C	-1.08670100	0.65628000	-1.25326300
C	-2.22931400	0.00490800	-0.53795900
C	-3.46246700	0.82965300	-0.95314200
C	-2.95197700	1.79811300	-2.03083600
C	-0.69065400	2.42780100	-2.94708800
N	-2.33485700	5.16791200	-0.76052300
C	-1.38014000	5.78673500	-1.52498300
O	-1.59003600	6.73036700	-2.26954000

N	-0.06673400	5.26705900	-1.44772700
C	0.32916700	4.14559300	-0.74546100
O	1.46734400	3.65215100	-0.87161500
C	-0.69865900	3.58568800	0.04517800
N	-0.44207300	2.37043200	0.73474700
C	-1.28194900	2.11130500	1.82874800
C	-0.89967400	1.26957300	2.86824000
C	-1.77453700	0.93491700	3.90597600
C	-1.30453000	0.04320700	5.02466100
C	-3.07548300	1.44632200	3.88372400
C	-4.07181600	1.05312900	4.94247600
C	-3.45206200	2.31445200	2.85162400
C	-2.57935500	2.66742300	1.82276000
N	-2.96454900	3.52707900	0.77628100
C	-1.96824700	4.14672500	0.00266800
C	-4.30431700	4.08012500	0.78252400
H	3.50286300	3.40915000	-2.00558700
H	4.18681000	1.88499300	-2.58202400
H	7.27470500	3.44927000	-1.36421800
H	7.76471800	2.92762800	1.04824700
H	2.61416500	1.82752300	0.00656300
H	2.27436200	1.06599800	2.37620900
H	6.38775800	1.90066400	3.32122500
H	4.12172500	1.11622300	3.98628400
H	1.85910600	-3.87736100	-0.11792700
H	-0.32841900	-3.05280600	0.78499500
H	-1.20600700	-4.06020600	-0.36285400
H	-0.21919800	-2.71674600	-0.94989300

H	1.70687100	-6.29215300	0.57423500
H	0.81160300	-5.26666800	1.70139500
H	-0.05927700	-6.20566800	0.48441200
H	-3.54261700	-5.54842800	-2.99170000
H	-4.48674400	-4.69737700	-1.76192600
H	-4.99468000	-2.52866300	-2.64130700
H	-1.01320800	-4.06354400	-3.05933700
H	-4.31476800	-0.44995700	-3.78633700
H	-0.31559900	-1.95570300	-4.16340300
H	-2.59057500	0.53580000	-5.03220000
H	3.70892300	-4.81512100	1.83753300
H	4.00083500	-5.18695100	0.13393500
H	4.05715600	-2.18345900	2.50149100
H	5.14748900	-0.21089200	1.43821400
H	6.19010200	-4.56104600	-1.56405100
H	7.46708800	-3.16969100	-3.16611000
H	6.72296200	0.37639600	-0.82415200
H	7.72913500	-0.74037800	-2.80806500
H	1.56446200	-3.04999700	1.95889800
H	-0.25243800	-3.87872800	3.41968300
H	-0.64507700	-2.21514600	2.90993800
H	-0.01860500	-2.52391000	4.53799500
H	2.13560200	-1.08086900	2.05387700
H	-7.29218000	0.65908300	1.09710700
H	-6.32385900	1.07761000	-0.31523100
H	-7.90271100	-2.14865900	-1.30529300
H	-6.51971800	-4.16555300	-0.71979500
H	-4.56492400	0.09271300	1.92792600

H	-2.78263600	-1.29298200	2.90727900
H	-4.16762500	-4.76956200	0.76157300
H	-2.56753000	-3.69351000	2.31943900
H	0.15263100	-0.29889300	0.92614300
H	0.72180000	0.27086400	-3.30779500
H	2.93077300	-0.89072500	-3.05621400
H	3.59329600	-1.73542500	-0.81245900
H	-3.85048300	1.38624300	-0.09898100
H	-4.25290600	0.17829300	-1.33095600
H	0.62650600	5.72646900	-2.02280000
H	0.10682900	0.85314900	2.87992600
H	-1.90669400	-0.87166500	5.09482000
H	-0.26255700	-0.24611100	4.87052700
H	-1.38247400	0.54968800	5.99291300
H	-4.27756500	-0.02377200	4.90423500
H	-5.01956600	1.57893900	4.80707600
H	-3.70218300	1.27501100	5.94912200
H	-4.45584800	2.72534400	2.86670600
H	-5.04281700	3.26940600	0.73514200
H	-4.39902200	4.73121100	-0.08352100
H	0.55351900	2.27904600	0.93342200
H	-2.06430200	0.00150000	0.54514100
H	-2.29486200	-1.04602900	-0.85624400
H	-3.15426700	2.85205700	-1.81440400
H	-3.32776900	1.57203000	-3.03507900
H	-1.14488800	3.42088400	-2.97166900
H	0.32537100	2.51279200	-2.55612900
H	-0.69916000	1.98962700	-3.94810500

H	5.01512900	3.40548500	-2.95182000
H	5.28853700	-5.42397200	1.31688000
H	2.17458400	-3.18392000	3.61292800
H	1.02345900	-4.82923900	-1.33334000
H	-2.80103200	-5.13647900	-1.44372400
H	-7.96523100	0.45355200	-0.52532800
H	-4.49344900	4.66860400	1.68922000

MS2 with cam-B3LYP

RC1

C	-1.78218167	2.06624603	0.02333004
H	-0.81717067	2.26309303	-0.45351296
H	-2.19440367	3.03471703	0.31233904
C	-1.57207167	1.23117803	1.23895704
N	-0.32828167	1.16422003	1.87150304
C	-0.41402967	0.46032103	3.01100404
H	0.35790533	0.29499903	3.74230404
N	-1.67567767	0.05633003	3.13057204
C	-2.40799867	0.50534403	2.04200804
H	-3.45002367	0.28539703	1.93312504
C	-2.98084467	-5.35279397	0.27936404
H	-4.03726067	-5.49150697	0.49876704
H	-2.57391967	-6.32615197	-0.00417096
C	-2.29390367	-4.79706697	1.47438904
N	-0.91322067	-4.78490797	1.60628604
C	-0.57877767	-4.09661797	2.72704904
H	0.43616933	-3.93688897	3.03676304
N	-1.67607267	-3.66599497	3.33279004

H	-2.00710567	-0.54518897	3.90722204
C	-2.75429967	-4.10008597	2.56175704
H	-3.77170567	-3.85923997	2.82500204
C	-0.27838567	2.56752103	5.49544004
H	-0.95647867	3.41681003	5.44359504
H	0.09385033	2.37155403	4.48557304
C	-1.05893567	1.38602903	6.01560504
O	-2.21156067	1.53130503	6.46380504
O	-0.45497567	0.19052303	5.97194204
C	0.94800633	-3.19063097	5.75006804
H	0.17744533	-2.55401297	5.30973204
H	0.96516133	-4.10990197	5.16663604
C	0.47112133	-3.49772497	7.11639304
N	-0.66958767	-2.84462097	7.58787104
C	-0.99830167	-3.39253597	8.75694204
H	-1.87002767	-3.15561397	9.35510704
N	-0.09752767	-4.35693797	9.05197904
H	-0.11247567	-4.89185797	9.91370904
C	0.84039533	-4.43665897	8.03478404
H	1.63581933	-5.15353797	8.04042704
C	-2.50052767	0.21925703	10.77428804
H	-2.18573167	-0.74628097	11.17430404
H	-3.58524167	0.14592403	10.69153504
C	-1.91582367	0.37185803	9.38516204
O	-1.22407067	1.32727103	9.02772504
O	-2.28512367	-0.65092697	8.56997004
C	-8.40730267	-1.92102397	12.73452304
H	-8.76056967	-2.84485997	13.20524704

H	-9.22837067	-1.56504197	12.10420604
C	-7.20579967	-2.16950397	11.85439004
C	-7.19396367	-1.70032197	10.53486004
H	-8.09646067	-1.24851497	10.13500004
C	-6.06463767	-1.81344097	9.72843604
H	-6.06876367	-1.47439497	8.70462904
C	-4.90455767	-2.40218297	10.23639704
O	-3.74937267	-2.53818497	9.47370204
H	-3.33193067	-1.69896897	9.06899204
C	-4.90517067	-2.88190197	11.53918804
H	-3.99092267	-3.29251197	11.94795004
C	-6.04537167	-2.78608897	12.32925704
H	-5.99249167	-3.16555097	13.34472504
N	-6.50909467	-12.75472697	9.84186704
C	-5.36346767	-1.67520297	5.84960704
O	-4.98087767	-1.21708197	6.93311804
N	-4.63442267	-8.72778797	7.60634704
N	-4.93155067	-2.93734097	5.45090804
C	-5.47550267	-9.95961897	7.42348704
C	-6.31679867	-12.00974397	8.54201504
C	-4.68192867	-7.71804597	6.46787004
C	-4.33540667	-3.87162497	6.42847404
C	-5.55244767	-10.71586397	8.74910704
C	-4.11698967	-6.37658597	6.92119204
C	-4.57399367	-5.29561297	5.93895804
C	-6.19787067	-0.88325497	4.89189904
Zn	-1.53088967	-1.18343397	6.83037304
O	-2.36760167	-1.85876397	5.02873204

H	-3.32355067	-2.11321397	5.04468404
H	-6.99886867	-13.65189597	9.63651504
H	-7.14398467	-12.26396297	10.49507504
H	-4.91730367	-8.24272097	8.48102304
H	-5.38821267	-3.37290997	4.64736204
H	-6.46737267	-9.63616697	7.10576704
H	-5.01945467	-10.55358697	6.63144304
H	-7.31601067	-11.83465797	8.14093604
H	-5.79811367	-12.69209697	7.86655104
H	-4.14506267	-8.16082597	5.63096204
H	-5.73820667	-7.62328697	6.21456704
H	-3.26399367	-3.68424597	6.53278104
H	-4.77008467	-3.69379697	7.41587404
H	-6.06174467	-10.09148197	9.49466204
H	-4.54639267	-10.93177897	9.12646904
H	-4.50409567	-6.13378097	7.92149704
H	-3.02573767	-6.41254697	6.99843804
H	-4.07855167	-5.44657197	4.97157404
H	-5.64953467	-5.42685697	5.78088304
H	-6.65312867	-1.50264397	4.11676504
H	-5.56945167	-0.13016097	4.40381004
H	-6.97411167	-0.35293397	5.44045804
H	-5.63722767	-12.96561497	10.32538404
H	-3.65903367	-9.00225597	7.80610704
H	-0.25628867	-5.19479797	0.94706504
H	0.56308533	1.51453203	1.51023204
H	-1.91579167	-2.61447097	4.44053404

TS1

C	0.39432177	1.02523658	0.00000000
H	-0.58170223	0.98712058	0.49201900
H	0.67030377	2.07730858	-0.03413600
C	0.27675977	0.49730358	-1.38715500
N	-0.89798623	0.64434758	-2.12501000
C	-0.74558323	0.13203158	-3.36157500
H	-1.43901523	0.14264258	-4.18965800
N	0.49239977	-0.34306242	-3.44319100
C	1.14393577	-0.13074742	-2.24105500
H	2.17482177	-0.38714442	-2.11017800
C	2.01105677	-4.27916942	-0.82532400
H	2.97343477	-3.90459742	-1.16814600
H	2.16918377	-5.33098542	-0.55362500
C	1.06806977	-4.26645642	-1.97810800
N	-0.29714723	-4.54327242	-1.87235000
C	-0.86076523	-4.62009242	-3.08165200
H	-1.90075623	-4.80056042	-3.27559200
N	0.10698477	-4.42260842	-3.98040500
H	0.87869077	-0.88404842	-4.31140300
C	1.31432577	-4.18147942	-3.32470500
H	2.21372577	-3.95595942	-3.87755100
C	-0.99669423	2.51618758	-5.53251500
H	-0.25649723	3.29348158	-5.34787200
H	-1.40030723	2.18751558	-4.57021100
C	-0.31127023	1.37042558	-6.23366300
O	0.79695377	1.53452258	-6.79525200
O	-0.89666123	0.18002558	-6.24501000

C	-2.05131723	-2.82640942	-5.81018800
H	-1.42630923	-2.01541742	-5.41535000
H	-2.17727623	-3.58572742	-5.03060900
C	-1.33899023	-3.44398442	-6.96742800
N	-0.49760723	-2.68048642	-7.77730600
C	-0.03106223	-3.46924742	-8.74252900
H	0.68477977	-3.19875642	-9.51087900
N	-0.52942023	-4.72500942	-8.57237600
H	-0.27580423	-5.53056642	-9.15124500
C	-1.35372523	-4.73531942	-7.45221600
H	-1.89236223	-5.61861842	-7.15632800
C	0.40187877	0.82474158	-11.29462600
H	-0.14833623	-0.00192542	-11.74742300
H	1.45660477	0.56014358	-11.38660900
C	0.04825577	0.84679058	-9.81684500
O	-0.60493523	1.75318258	-9.26839400
O	0.48841777	-0.24875042	-9.20620600
C	7.50157577	-1.49179642	-12.54022300
H	8.08758177	-2.32140142	-12.95278800
H	8.18262277	-0.92840442	-11.89559100
C	6.34412177	-1.99511942	-11.71146300
C	6.12520577	-1.52160942	-10.41021700
H	6.87459277	-0.89187442	-9.94317200
C	4.96458977	-1.84609042	-9.70695700
H	4.80708877	-1.51291042	-8.68713600
C	3.98914377	-2.63985342	-10.31497200
O	2.79177677	-2.93837242	-9.68059000
H	2.64770777	-2.39600142	-8.85807700

C	4.21350677	-3.15693342	-11.58206700
H	3.44168777	-3.74908942	-12.05253400
C	5.38543277	-2.85228042	-12.26159900
H	5.51424677	-3.25946642	-13.25704800
N	2.80215077	-14.39761542	-7.43660800
C	2.84097877	-2.03271742	-6.25709900
O	2.44693177	-1.67030842	-7.45072400
N	2.80727177	-9.37436242	-7.70916500
N	3.07681577	-3.39084342	-6.02200100
C	3.23545377	-10.66762842	-7.08907200
C	2.95845477	-13.05096542	-6.77647800
C	3.39278277	-8.18663842	-6.95327900
C	2.56319777	-4.43098742	-6.89887900
C	2.59383677	-11.90066742	-7.71314300
C	2.74046777	-6.89348042	-7.38940500
C	3.18586377	-5.75498442	-6.48165000
C	3.73144977	-1.11542342	-5.46663300
Zn	0.47068277	-0.87577542	-7.37377900
O	1.25652577	-1.73267642	-5.39836900
H	0.81833977	-2.60629642	-5.38146400
H	3.20648377	-15.11977742	-6.82002900
H	3.40000077	-14.41109542	-8.29137200
H	3.10319677	-9.32266542	-8.70163300
H	3.82927677	-3.65404442	-5.38506700
H	4.32125177	-10.70933242	-7.19567200
H	2.98307377	-10.58941242	-6.02911000
H	4.01437777	-12.99545442	-6.49502000
H	2.35474377	-13.04516942	-5.86922000

H	3.21661677	-8.39651942	-5.89911800
H	4.46250377	-8.20142342	-7.15514000
H	1.46337877	-4.50915542	-6.81415900
H	2.78136877	-4.21574042	-7.95278800
H	2.99618777	-12.06440842	-8.72030100
H	1.50719077	-11.79180142	-7.79520900
H	3.01017877	-6.65497742	-8.42358600
H	1.64571077	-6.99200042	-7.33341500
H	2.90892177	-5.98694942	-5.44523700
H	4.27447577	-5.65923542	-6.52249800
H	3.85289077	-1.49086442	-4.44513900
H	3.29119777	-0.12699942	-5.44701600
H	4.71266677	-1.06574742	-5.94402600
H	1.84780977	-14.62943442	-7.70591800
H	1.77790477	-9.29019542	-7.73008400
H	-0.81320123	-4.70823942	-1.00873100
H	-1.76717923	1.08952658	-1.81260900
H	-0.04121323	-4.46433442	-4.99124500

PC1

C	-0.31525746	2.01872751	-0.03055528
H	-1.20200146	1.86102551	0.59246172
H	-0.16708446	3.09815051	-0.06800028
C	-0.55859246	1.47910651	-1.39495328
N	-1.80173946	1.57384051	-2.01549228
C	-1.76360146	1.01366151	-3.23676128
H	-2.59221546	0.93712351	-3.91641128
N	-0.52237546	0.57816751	-3.43667528

C	0.23560054	0.84028951	-2.31169728
H	1.27284554	0.58012951	-2.26590628
C	1.16795054	-3.35484149	-0.52752528
H	2.17764154	-3.23026349	-0.91232428
H	1.07169654	-4.40068549	-0.22347028
C	0.23740254	-3.08041149	-1.65280228
N	-1.11562946	-3.42118449	-1.67787828
C	-1.66062446	-3.07198649	-2.85871128
H	-2.68329946	-3.21361049	-3.15323728
N	-0.69855946	-2.52231249	-3.58992028
H	-0.21389746	0.04839751	-4.31876028
C	0.48618454	-2.52808149	-2.87833828
H	1.39746954	-2.21320849	-3.34984628
C	-2.15535846	2.79819251	-6.23521428
H	-1.47410046	3.62361051	-6.03050628
H	-2.41018446	2.30349351	-5.30098428
C	-1.47878046	1.85265051	-7.20408128
O	-0.76035946	2.28536351	-8.09849128
O	-1.73608246	0.51337151	-7.04862228
C	-3.18449446	-2.66781249	-6.53601728
H	-2.38167546	-1.94894949	-6.32869128
H	-3.07136346	-3.49898549	-5.82858428
C	-2.96119946	-3.17691649	-7.91573828
N	-2.27683446	-2.38161649	-8.83673828
C	-2.10967846	-3.09650949	-9.94403128
H	-1.54655446	-2.80526549	-10.81409028
N	-2.68255146	-4.31400449	-9.78031728
H	-2.54603846	-5.06738849	-10.45541628

C	-3.22478146	-4.38873249	-8.49806428
H	-3.68103646	-5.28434949	-8.11838228
C	-1.75444446	1.03081851	-12.30520628
H	-2.47170046	0.26940451	-12.62652728
H	-0.77177246	0.66234651	-12.59780128
C	-1.81299446	1.08655651	-10.79002328
O	-2.47170246	1.92395351	-10.15035228
O	-1.11793146	0.08600251	-10.24091628
C	4.67054054	-1.65445549	-13.61781028
H	5.23986554	-2.54409149	-13.90742128
H	5.39203154	-0.98110749	-13.14530928
C	3.60952554	-2.00608149	-12.59913928
C	3.57718854	-1.33271649	-11.36640728
H	4.32440854	-0.57509549	-11.16305328
C	2.65446354	-1.66627249	-10.37822328
H	2.66382554	-1.20416149	-9.39756228
C	1.71336054	-2.66836249	-10.62289228
O	0.78149154	-3.03382749	-9.65987428
H	0.81280254	-2.48012049	-8.81299828
C	1.70454154	-3.32597249	-11.84822528
H	0.93344354	-4.07479249	-12.04498228
C	2.65624254	-3.00826449	-12.81672728
H	2.63658554	-3.56981849	-13.74292128
N	1.25816954	-11.47023249	-7.01027728
C	0.90101854	-1.42527049	-6.34870028
O	0.54617654	-1.44782849	-7.68946328
N	1.23287854	-7.34216549	-9.82384228
N	1.04726354	-2.74541149	-5.75716028

C	1.99456954	-8.18943249	-8.83812328
C	1.94039654	-10.16180249	-7.33448828
C	1.24622354	-5.84303649	-9.48219128
C	0.33335054	-3.87259649	-6.33138928
C	1.25380754	-9.48666449	-8.51732628
C	0.46341054	-5.59989649	-8.19521528
C	1.24733154	-4.71844249	-7.22235028
C	2.18005454	-0.63758249	-6.11746628
Zn	-1.10192746	-0.76429849	-8.53246028
O	-0.22071146	-0.78415849	-5.60227428
H	-0.89440146	-0.18434949	-6.20917628
H	1.75197854	-11.96993349	-6.25323428
H	1.31989854	-12.09713449	-7.84011428
H	1.61357554	-7.46691249	-10.77468028
H	1.99245654	-2.98356249	-5.44623528
H	2.97480054	-8.38886949	-9.27400628
H	2.12063854	-7.58269749	-7.94032328
H	2.98300854	-10.40327549	-7.55280528
H	1.89621254	-9.54336149	-6.43699328
H	2.29112754	-5.54761149	-9.42369728
H	0.79231254	-5.32904349	-10.32891428
H	-0.12220446	-4.49414749	-5.54461228
H	-0.47846646	-3.47492649	-6.95004528
H	1.24557154	-10.14603949	-9.39349028
H	0.21266854	-9.26385349	-8.25489028
H	-0.47577546	-5.10534549	-8.46273128
H	0.19477554	-6.55368249	-7.71395928
H	1.93233354	-5.32579749	-6.61189628

H	1.86326054	-4.02927349	-7.80446728
H	2.36765454	-0.49577849	-5.04572228
H	2.10993754	0.32158451	-6.61191428
H	3.01742554	-1.19141949	-6.55000628
H	0.27530654	-11.35369349	-6.75501728
H	0.24002954	-7.62698049	-9.89237128
H	-1.61503846	-3.90696049	-0.93480728
H	-2.67137846	1.95518951	-1.63093428
H	-0.74328546	-2.07048749	-4.53257628

MS3 with M06-2X

RC2

C	-1.46528668	1.34032777	-0.07513084
H	-2.35059868	1.18237777	0.55048316
H	-1.32091868	2.42131977	-0.10781484
C	-1.70671368	0.81277077	-1.44350984
N	-2.93787768	0.94754577	-2.08439484
C	-2.88573668	0.41926777	-3.31859984
H	-3.69664268	0.38613577	-4.02222684
N	-1.65252568	-0.03997323	-3.50239984
C	-0.91184468	0.18046177	-2.35851784
H	0.11814032	-0.09842523	-2.30202784
C	-0.01468368	-4.07820923	-0.44745884
H	1.02765332	-4.04073223	-0.75855984
H	-0.19872968	-5.09490323	-0.09138884
C	-0.83739468	-3.82814623	-1.65553084
N	-2.21096468	-3.99699923	-1.76673484
C	-2.58559368	-3.75022223	-3.05249784

H	-3.60073368	-3.79944023	-3.40283884
N	-1.52172968	-3.43747123	-3.77338984
H	-1.30585468	-0.48516623	-4.39254884
C	-0.43150868	-3.49450723	-2.91841584
H	0.56769332	-3.33492723	-3.28521284
C	-3.28271768	2.55399877	-6.27006584
H	-2.61823968	3.38983477	-6.04783984
H	-3.48651168	2.00365077	-5.35170984
C	-2.62279468	1.68568477	-7.31558884
O	-1.96321568	2.17953977	-8.22403384
O	-2.82562468	0.33001177	-7.20302484
C	-4.35812568	-2.96359323	-6.80196384
H	-3.55075768	-2.26861023	-6.54817784
H	-4.21736768	-3.85097723	-6.17459084
C	-4.19112268	-3.34291023	-8.22825884
N	-3.47729368	-2.50819923	-9.09117284
C	-3.34839568	-3.14106323	-10.25251784
H	-2.78606368	-2.80085823	-11.10422584
N	-3.97599968	-4.33754923	-10.18137184
H	-3.88200568	-5.03970923	-10.91379084
C	-4.51416968	-4.48611123	-8.90532284
H	-5.01432868	-5.38157223	-8.58807284
C	-2.96691468	1.05135177	-12.42637584
H	-3.64022268	0.27953577	-12.81210584
H	-1.96305768	0.75372477	-12.73078884
C	-3.05064368	1.00754377	-10.91574384
O	-3.71944068	1.79749177	-10.22859684
O	-2.36025368	-0.02902023	-10.42073284

C	3.60261832	-1.51306323	-14.09138484
H	4.16609332	-2.36690823	-14.48451984
H	4.34259932	-0.86427123	-13.61296384
C	2.60074932	-1.95616623	-13.05250784
C	2.56794432	-1.32392023	-11.80059784
H	3.29494132	-0.55232523	-11.58126984
C	1.66524732	-1.71047323	-10.81658484
H	1.68540232	-1.28321023	-9.82185384
C	0.75022332	-2.72664223	-11.08241584
O	-0.16226768	-3.14611123	-10.12073184
H	-0.23453668	-2.55785123	-9.31835184
C	0.75571432	-3.36111323	-12.31656184
H	0.00794332	-4.13047323	-12.51689084
C	1.68390632	-2.98651723	-13.28504884
H	1.67417832	-3.51965923	-14.22818084
N	0.28612532	-11.88876123	-7.69964884
C	-0.21778868	-1.61703023	-6.74926584
O	-0.64002768	-1.63654823	-8.03627984
N	0.29077732	-7.62554323	-10.33410784
N	-0.33369568	-3.10782723	-6.10162684
C	1.03650532	-8.52413023	-9.37690584
C	0.94976832	-10.55272623	-7.94655784
C	0.34744732	-6.14166023	-9.93609984
C	-0.92284668	-4.16784123	-6.95313784
C	0.30543432	-9.84292323	-9.13335684
C	-0.51783968	-5.90808123	-8.70527284
C	0.13675732	-4.92235823	-7.73600884
C	1.24008232	-1.23982623	-6.57609084

Zn	-2.36137468	-0.89627323	-8.73409484
O	-1.06149268	-0.84137623	-5.92272984
H	-1.84217168	-0.32706523	-6.43669784
H	0.76939832	-12.40337723	-6.94653684
H	0.39515732	-12.47834323	-8.55079484
H	0.66986932	-7.72507423	-11.28717284
H	0.60437932	-3.41060923	-5.79219884
H	2.02961432	-8.68628623	-9.79859484
H	1.13819732	-7.96523723	-8.44770084
H	2.00593732	-10.76706223	-8.12453884
H	0.85502532	-9.97318023	-7.02731884
H	1.39515132	-5.91113923	-9.77855884
H	-0.01188868	-5.56510723	-10.78767584
H	-1.49367768	-4.83956223	-6.30302584
H	-1.60806868	-3.68155223	-7.64543484
H	0.34289032	-10.47089123	-10.03102084
H	-0.75025968	-9.64859323	-8.90985684
H	-1.48517568	-5.52054123	-9.03741084
H	-0.72138068	-6.85380423	-8.18231284
H	0.82057732	-5.45848523	-7.06412484
H	0.72374732	-4.20951023	-8.31545384
H	1.52571732	-1.27714423	-5.51840284
H	1.40757832	-0.23617923	-6.94956184
H	1.86691832	-1.93715023	-7.13988684
H	-0.70651068	-11.80739623	-7.47417784
H	-0.70801868	-7.88178923	-10.41126984
H	-2.82953468	-4.35923323	-1.04606584
H	-3.81044668	1.31751477	-1.69816384

H	-0.94365268	-3.06321323	-5.21424684

TS2

C	1.30467598	0.35452391	-0.13269759
H	0.42921298	0.16155491	0.49677741
H	1.41984298	1.43959691	-0.14370959
C	1.06085898	-0.14674709	-1.51110159
N	-0.18988702	-0.04941609	-2.11925659
C	-0.13783002	-0.52794809	-3.37483759
H	-0.96839802	-0.58343409	-4.05321959
N	1.11171698	-0.91469109	-3.60558059
C	1.86440398	-0.70374409	-2.46793959
H	2.90611098	-0.94223909	-2.44041959
C	2.87288798	-5.01542409	-0.57703159
H	3.89784398	-4.91541609	-0.92919259
H	2.75589698	-6.04708609	-0.23597459
C	1.98445898	-4.77590709	-1.73936859
N	0.61877598	-5.01664709	-1.78497159
C	0.16587098	-4.73500509	-3.04054459
H	-0.85956902	-4.82749109	-3.34851459
N	1.16639298	-4.33067909	-3.80061259
H	1.46642898	-1.30352709	-4.53244059
C	2.30233798	-4.36510609	-3.00516659
H	3.27056598	-4.14103609	-3.41929359
C	-0.51925602	1.73079691	-6.35600559
H	0.12892398	2.56783891	-6.09356359
H	-0.70221702	1.12529491	-5.46834159
C	0.14682998	0.94472791	-7.45347459

O	0.77530398	1.46729391	-8.35077659
O	-0.03589902	-0.45581709	-7.40079859
C	-1.65355402	-3.76492209	-6.95376259
H	-0.83463902	-3.07983309	-6.71382259
H	-1.46790702	-4.67646809	-6.37499659
C	-1.58426902	-4.07604409	-8.40541759
N	-0.71775902	-3.36206609	-9.23412659
C	-0.75247602	-3.92292209	-10.44060259
H	-0.14913702	-3.64887809	-11.28974359
N	-1.63517102	-4.94627909	-10.42880459
H	-1.76509302	-5.55675509	-11.23024959
C	-2.17565602	-5.05718909	-9.15443859
H	-2.87842902	-5.82207109	-8.88931059
C	-0.11375202	0.33411591	-12.46193359
H	-0.66678702	-0.49612709	-12.90839759
H	0.92919998	0.16819891	-12.73047259
C	-0.25365402	0.21496991	-10.96207659
O	-0.96296902	0.95377091	-10.25798359
O	0.46820298	-0.81055409	-10.49339859
C	6.35360998	-2.12166809	-14.36020959
H	6.88627498	-2.98106309	-14.78333059
H	7.12628398	-1.48258509	-13.92209359
C	5.40400798	-2.55511509	-13.26986159
C	5.47595898	-1.96092809	-12.00157959
H	6.24985698	-1.23015209	-11.80601959
C	4.61652598	-2.33457709	-10.97398259
H	4.72381498	-1.93953109	-9.97068959
C	3.64274798	-3.30256009	-11.20986559

O	2.77348198	-3.71838409	-10.20953159
H	2.77237898	-3.17136009	-9.37899859
C	3.54434898	-3.89611109	-12.46040359
H	2.75002598	-4.62315909	-12.63543259
C	4.42732898	-3.53516209	-13.47385359
H	4.33414098	-4.03925009	-14.42853959
N	3.08631498	-12.55846409	-8.00833159
C	2.57633798	-2.17550009	-6.82575359
O	2.25193498	-2.27873809	-8.11570759
N	3.12085298	-8.27021109	-10.61606759
N	2.47026898	-4.01787809	-6.16025759
C	3.86214698	-9.20229709	-9.68654159
C	3.75841198	-11.22667909	-8.25579259
C	3.21050598	-6.80267509	-10.16544959
C	1.89905998	-4.99633409	-7.08616559
C	3.10595098	-10.50436209	-9.43152759
C	2.33048098	-6.61050509	-8.93964159
C	2.97172898	-5.68281409	-7.91153459
C	4.04133798	-1.96582209	-6.53937759
Zn	0.52173698	-1.86259909	-8.93242759
O	1.69884798	-1.54943209	-6.04532859
H	0.61467698	-0.92507809	-6.69735059
H	3.57281798	-13.08421509	-7.26496059
H	3.17621598	-13.14347209	-8.86458859
H	3.48681898	-8.35423109	-11.57519859
H	3.40219698	-4.32211209	-5.84406659
H	4.84043098	-9.38617709	-10.13342559
H	4.00248098	-8.65467609	-8.75541659

H	4.81045398	-11.44903209	-8.44767459
H	3.67963698	-10.65129409	-7.33241659
H	4.25846398	-6.60482109	-9.97564059
H	2.88072698	-6.18109109	-10.99734959
H	1.30448098	-5.73721809	-6.53247559
H	1.22412998	-4.46744809	-7.76344059
H	3.11397198	-11.13295109	-10.32939859
H	2.05931798	-10.28744709	-9.18820659
H	1.37261398	-6.19596709	-9.26658959
H	2.10956298	-7.57671009	-8.46267059
H	3.65829798	-6.25119909	-7.26874259
H	3.55609398	-4.92723909	-8.43674459
H	4.24639898	-2.09848609	-5.47297559
H	4.33618198	-0.95895809	-6.82538059
H	4.63648798	-2.68059109	-7.11009559
H	2.09775998	-12.46868509	-7.76786159
H	2.11340298	-8.49436409	-10.67882859
H	0.06147498	-5.44231909	-1.05038059
H	-1.06794102	0.27135791	-1.70319459
H	1.84707298	-3.94504109	-5.32861259

PC2

C	-1.78204666	1.57628852	-0.06461334
H	-2.62708766	1.47458252	0.62424366
H	-1.55264866	2.64257752	-0.10153034
C	-2.16885066	1.07963452	-1.41157734
N	-3.45096466	1.26666652	-1.92587734

C	-3.54586466	0.71594252	-3.15089034
H	-4.44194566	0.68323452	-3.74298434
N	-2.35926366	0.20458352	-3.45564434
C	-1.49533266	0.40227952	-2.39587634
H	-0.47829966	0.06196252	-2.43384434
C	-0.17988466	-3.87692548	-0.80713834
H	0.85678334	-3.71107748	-1.09302234
H	-0.24768966	-4.92370248	-0.48889434
C	-0.99422566	-3.70707948	-2.04232134
N	-2.36087366	-3.91336348	-2.15795534
C	-2.70837766	-3.79512248	-3.47717134
H	-3.72377266	-3.88634248	-3.81415234
N	-1.64184266	-3.53502248	-4.22316834
H	-2.11110666	-0.26748348	-4.40345234
C	-0.57162966	-3.48196048	-3.33134734
H	0.43405434	-3.31751748	-3.67665734
C	-3.88460666	1.68803852	-6.67027634
H	-3.21195266	2.48826952	-6.36275234
H	-4.22057066	1.12525352	-5.79745134
C	-3.16159666	0.79147552	-7.64673134
O	-2.32043166	1.20163352	-8.41833434
O	-3.63205466	-0.52772948	-7.67802434
C	-4.67578666	-3.68262948	-6.56138034
H	-3.82836966	-2.99212748	-6.48459234
H	-4.54141966	-4.41876648	-5.76215634
C	-4.58327566	-4.36802548	-7.87631034
N	-3.64554066	-3.94972448	-8.81354334
C	-3.70880966	-4.78057248	-9.85016634

H	-3.07934566	-4.75778048	-10.72522634
N	-4.67647266	-5.70128748	-9.62865234
H	-4.86891266	-6.45244248	-10.28352834
C	-5.24635466	-5.45649548	-8.38833334
H	-6.00807666	-6.08678648	-7.97571234
C	-3.08464366	-0.75538148	-12.44302634
H	-3.84093766	-1.50353248	-12.69674434
H	-2.12243966	-1.18430048	-12.71778534
C	-3.10890766	-0.55608648	-10.93867634
O	-3.79911566	0.32840152	-10.40725234
O	-2.31518366	-1.42661248	-10.30025134
C	3.33540434	-3.03949948	-13.09484634
H	3.94480234	-3.93245948	-13.27224634
H	4.02202434	-2.28080348	-12.70791034
C	2.28521734	-3.31456848	-12.04196734
C	2.26430934	-2.55698248	-10.85837034
H	3.00508434	-1.77904648	-10.72008434
C	1.33707734	-2.81026348	-9.84828634
H	1.32602734	-2.26178848	-8.91374334
C	0.39455134	-3.82173248	-10.02370034
O	-0.61232066	-4.02263948	-9.06449834
H	-0.31480166	-4.30531248	-8.06015434
C	0.39550534	-4.58590248	-11.18383834
H	-0.36689366	-5.35395148	-11.31317134
C	1.34259234	-4.34195948	-12.17721934
H	1.32231234	-4.97363048	-13.05685534
N	0.30567034	-13.58939848	-6.34947634
C	-0.91274166	-1.32222248	-6.28088434

O	-0.98952166	-1.97518148	-7.37504834
N	-0.11090066	-9.39582848	-9.08505134
N	-0.14447466	-4.61544948	-6.62174134
C	0.18878334	-10.08723848	-7.78436634
C	0.68885734	-12.14968148	-6.57833534
C	0.61424734	-8.05191448	-9.28017734
C	-0.63460366	-5.94867448	-6.22520634
C	0.06867734	-11.60567048	-7.86491534
C	0.07758334	-6.93456448	-8.41266834
C	0.20634234	-7.03575648	-6.88881934
C	0.45162734	-1.24185048	-5.64676834
Zn	-2.09974066	-2.67152448	-8.86802334
O	-1.90782466	-0.71170548	-5.76106534
H	-3.41273866	-0.90747648	-6.78346334
H	0.82736034	-13.96513348	-5.54045534
H	0.62288334	-14.14973148	-7.16945634
H	0.17035334	-9.99232148	-9.87916734
H	0.82831134	-4.51570648	-6.29878434
H	1.19832034	-9.78400148	-7.49946334
H	-0.51420666	-9.69912748	-7.05135534
H	1.77965934	-12.12514348	-6.62655834
H	0.36563934	-11.59101748	-5.70049334
H	1.66437034	-8.28027348	-9.11758934
H	0.45737334	-7.79672348	-10.33056434
H	-0.62545966	-6.06563948	-5.13670434
H	-1.67564966	-6.03256048	-6.55671634
H	0.60139734	-12.00233748	-8.73706234
H	-0.98433766	-11.89707048	-7.95172134

H	0.60403334	-6.04345448	-8.76708434
H	-0.97203766	-6.74216248	-8.66945334
H	-0.15469666	-7.98670748	-6.49562534
H	1.25599234	-6.94599348	-6.58812034
H	0.41131234	-0.76916148	-4.66572034
H	1.13249534	-0.68331248	-6.28928934
H	0.86661434	-2.25036748	-5.53843134
H	-0.69633766	-13.72096148	-6.21558834
H	-1.12595266	-9.21822248	-9.18433634
H	-2.99538666	-4.19956048	-1.41625834
H	-4.27951066	1.63981152	-1.45737234
H	-0.71065066	-3.90884148	-6.13986634
